# A Catenane Assembled through a Single Charge-Assisted Halogen Bond**

**DOI:** 10.1002/anie.201300464

**Published:** 2013-03-11

**Authors:** Lydia C Gilday, Thomas Lang, Antonio Caballero, Paulo J Costa, Vítor Félix, Paul D Beer

**Affiliations:** Inorganic Chemistry Laboratory, Department of Chemistry, University of OxfordSouth Parks Road, Oxford OX1 3QR (UK); Departamento de Química, CICECO and Secção Autónoma de Ciências da SaúdeUniversidade de Aveiro, 3810-193 Aveiro (Portugal)

**Keywords:** catenanes, halogen bonding, supramolecular chemistry, template synthesis

Interlocked molecules have captured chemists’ imagination owing to their nontrivial topology and the promise of their potential nanotechnological uses as molecular machines[1] and in chemical sensor applications.[2] The synthesis of such sophisticated architectures is a challenge, and as a consequence this has necessitated the implementation of imaginative cation,[3] anion,[4] and neutral[5] templation methodologies, which use a combination of complementary Lewis acid–base, electrostatic, and hydrogen-bonding interactions for component assembly. Halogen bonding (XB) is the attractive highly directional, noncovalent interaction between an electron-deficient halogen atom and a Lewis base.[6] The scope of XB in solid-state crystal engineering has been intensively explored for a number of years,[7] however, in spite of the complementary analogy to ubiquitous hydrogen bonding, it is only in recent times that investigations into the application of solution-phase XB interactions to molecular recognition processes, self-assembly, and catalysis have resulted in this field developing rapidly.[8] Indeed, in conjunction with anion templation, we have used XB to assemble interpenetrated and interlocked molecular frameworks.[8b–d]

By incorporating a suitable neutral Lewis base XB acceptor, such as pyridine,[7b, 9] into a macrocyclic framework, we demonstrate herein that a single charge-assisted XB interaction can be utilized for pseudorotaxane assembly and, importantly, in the synthesis of a novel interlocked catenane.

**Figure 1:**
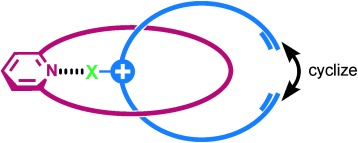
Cartoon of orthogonal halogen-bond-templated pseudorotaxane species. X=halogen atom.

**Scheme 1:**
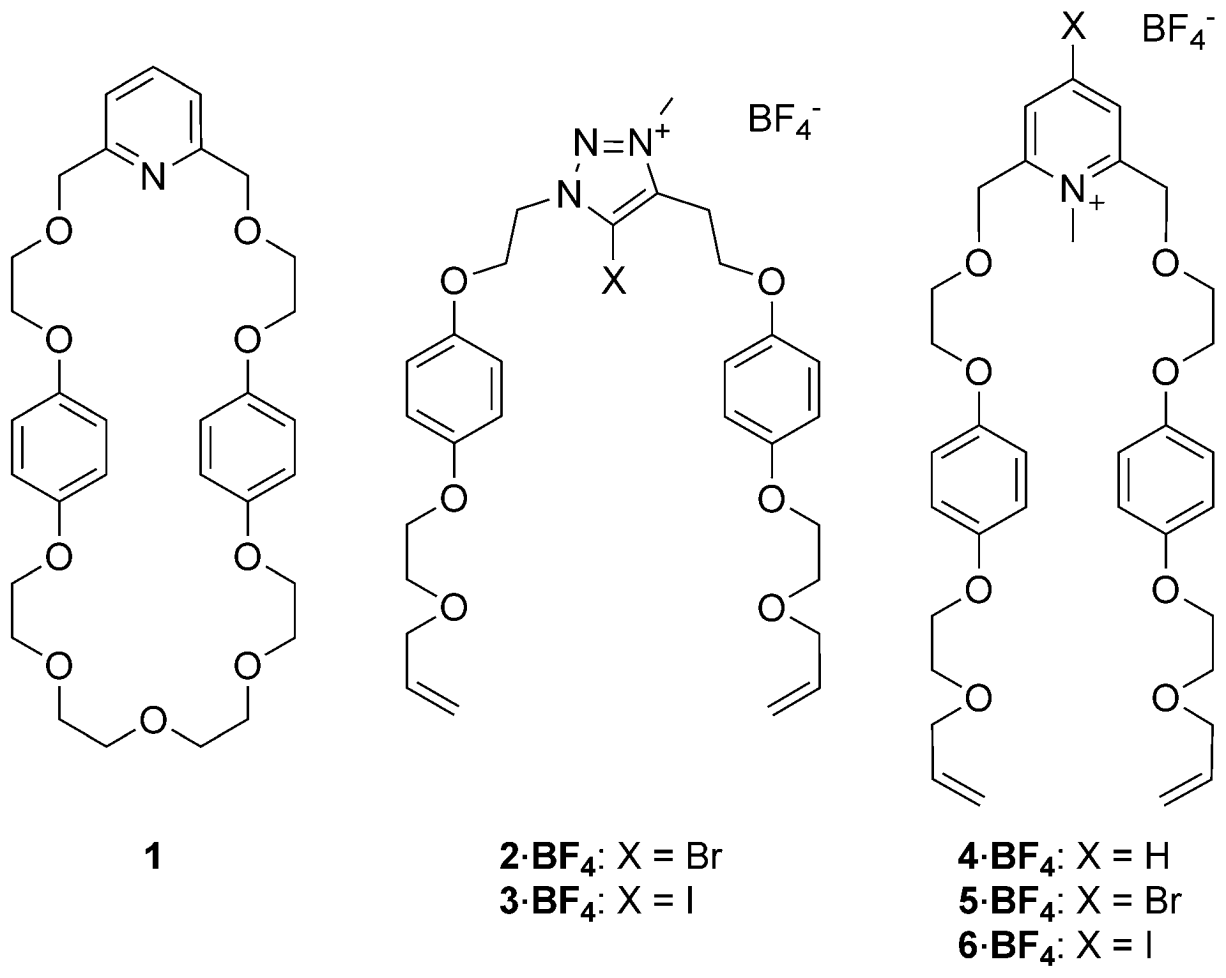
Structures of macrocycle 1 and threading components 2–6⋅BF_4_.

The synthetic strategy employed to construct the target catenane is outlined in Figure 1. An acyclic positively charged XB-donor component threads through a complementary XB-accepting pyridine-containing macrocycle, thereby forming an orthogonal interpenetrative assembly that is stabilized by a charge-assisted XB interaction. In addition to the incorporated pyridine XB-acceptor motif, the macrocycle component **1** contains electron-rich hydroquinone groups to facilitate supplementary secondary aromatic donor–acceptor interactions with electron-deficient positively charged heterocyclic XB-donor threading derivatives. A range of bromo- and iodo-functionalized triazolium and pyridinium compounds were chosen as potential threading components, containing terminal vinyl functional groups, which after successful pseudorotaxane assembly with macrocycle **1**, could be used for catenane synthesis through ring-closing metathesis (RCM) cyclization (Scheme 1). Macrocycle **1** and halo-functionalized triazolium and pyridinium derivatives were prepared by using multistep synthetic procedures described in Schemes S1–S3 in the Supporting Information.

**Scheme 2:**
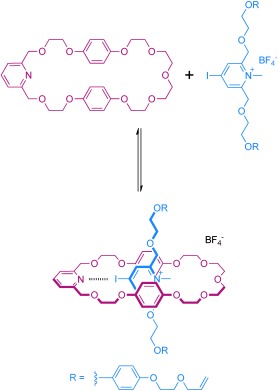
Pseudorotaxane assembly between macrocycle 1 and iodopyridinium threading component 6⋅BF_4_.

To establish evidence for interpenetrative assembly in solution (Scheme 2) and also to determine the strength of any association between respective macrocycle XB-acceptor and potential XB-donor heterocycle thread components, the pseudorotaxane assembly between macrocycle **1** and halo-functionalized triazolium and pyridinium compounds **2⋅BF_4_**–**6⋅BF_4_** in CD_2_Cl_2_ solution was investigated by using ^1^H NMR spectroscopy.

Upon addition of thread components **3⋅BF_4_**, **5⋅BF_4_**, and **6⋅BF_4_** to macrocycle **1**, the signals corresponding to the macrocycle hydroquinone protons were shifted upfield, which is attributed to aromatic donor–acceptor interactions with the respective electron-deficient heterocyclic aromatic surface.

These diagnostic changes in the ^1^H NMR spectrum of the macrocycle are highly indicative of successful formation of an interpenetrated species by threading through the annulus of the macrocycle component. The observed chemical shift perturbations of the hydroquinone protons with ten equivalents of thread component (Table 1) reveal the iodopyridinium derivative **6⋅BF_4_** causes the largest magnitude of perturbation; the chemical shift perturbation diminishes with the XB donor capability of the substituent X, as observed for **6⋅BF_4_**>**5⋅BF_4_**. Furthermore, these Δ*δ*_ppm_ values are greater for the pyridinium species than for the triazolium analogues. Notably, no significant evidence of interpenetration was observed with the bromotriazolium **2⋅BF_4_** derivative.

**Table 1:** Observed chemical shift perturbations (Δ*δ*) of hydroquinone protons and association constants (*K*_a_) for macrocycle 1 with thread components 3⋅BF_4_–6⋅BF_4_. Estimated standard errors are given in parentheses.

	Thread	3⋅BF_4_	4⋅BF_4_	5⋅BF_4_	6⋅BF_4_	
	Δ*δ* [ppm][a]	0.03[b]	0.03	0.07	0.10	
	*K*_a_ [m^−1^]	55(3)	30(2)	80(2)	180(20)	

[a] After ten equivalents.

[b] After five equivalents. CD_2_Cl_2_, 293 K.

**Figure 2:**
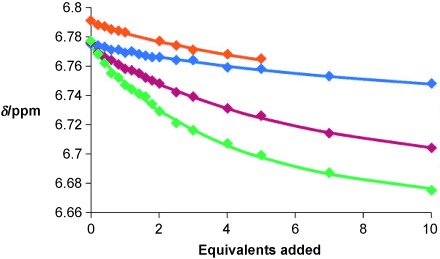
Titration curves for pseudorotaxane assembly between macrocycle 1 and thread components 3⋅BF_4_ (orange), 4⋅BF_4_ (blue), 5⋅BF_4_ (red), and 6⋅BF_4_ (green).

Quantitative analysis of the pseudorotaxane assembly process was achieved by monitoring the macrocycle’s hydroquinone protons as a function of the concentration of the thread component, and winEQNMR2[10] analysis of the titration data (Figure 2) gave 1:1 stoichiometric association constants for pseudorotaxane formation (Table 1). Importantly, the most stable interpenetrative assembly with macrocycle **1** is found with the iodopyridinium thread **6⋅BF_4_** with an association constant value (*K*_a_=180 m^−1^) more than double that of the pseudorotaxane assembly observed with bromopyridinium thread **5⋅BF_4_** (*K*_a_=80 m^−1^). This can be attributed to the greater halogen-bond-donor ability of the more polarizable iodine substituent as compared to bromine. Notably, the interpenetrative assembly with the corresponding protic pyridinium analogue **4⋅BF_4_** is significantly weaker (*K*_a_=30 m^−1^), thus highlighting the essential contribution of the XB interaction in stabilizing the overall pseudorotaxane assembly process.

Table 1 shows that although the iodotriazolium compound **3⋅BF_4_** forms a measurable pseudorotaxane association with the pyridine macrocycle **1**, the extremely small chemical shift perturbations noted in the titration experiment with the bromotriazolium analogue **2⋅BF_4_** suggest interpenetration is not occurring. The reduced supplementary secondary aromatic donor–acceptor interactions of the triazolium motif with the macrocycle’s hydroquinone groups in comparison with pyridinium will also be a contributing factor.

**Scheme 3:**
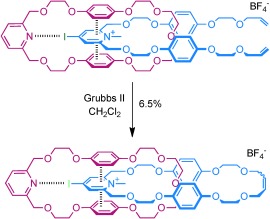
Synthesis of a novel halogen-bond-templated catenane 7⋅BF_4_.

Encouraged by the favorable association between macrocycle **1** and iodopyridinium thread **6⋅BF_4_**, the synthesis of a novel [2]catenane using a RCM clipping reaction in the presence a Grubbs catalyst was undertaken (Scheme 3).

Macrocycle **1** and iodopyridinium thread **6⋅BF_4_** (1.1 equiv) in anhydrous CD_2_Cl_2_ solution were stirred at room temperature under N_2_ in the presence of Grubbs(II) catalyst (10 wt %). After purification using repeated preparative thin layer chromatography, the target [2]catenane **7⋅BF_4_** was isolated in 6.5 % yield.

Evidence for the successful formation of a halogen-bonded [2]catenane species is provided by electrospray mass spectrometry and ^1^H NMR spectroscopy.

The cationic molecular ion peak is readily observed in the mass spectrum, which suggests the presence of a single interlocked species, and the high-resolution mass spectrum reveals the isotopic distribution of the [2]catenane is in good agreement with the simulated spectrum (see Figure S1 in the Supporting Information).

**Figure 3:**
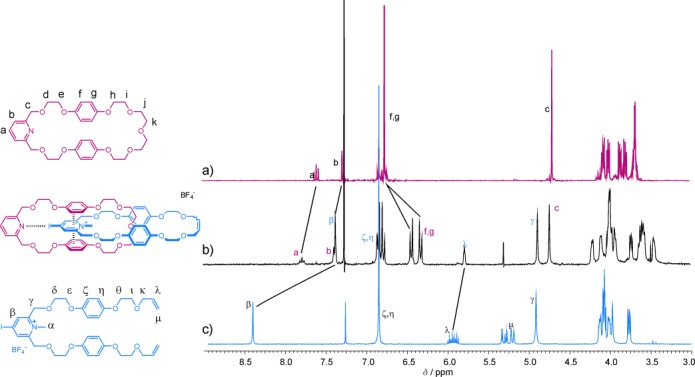
^1^H NMR spectra of a) pyridine macrocycle 1, b) halogen-bonded [2]catenane 7⋅BF_4_, c) iodopyridinium RCM precursor 6⋅BF_4_ (300 MHz, CDCl_3_, 298 K).

The ^1^H NMR spectrum of the [2]catenane and those of the component macrocycle and vinyl-appended precursor are shown in Figure 3. The disappearance of the multiplet μ (vinylic protons) and the convergence of multiplet λ into a pseudo singlet in the catenane spectrum are attributed to cyclization of the acyclic precursor. Most importantly, the upfield shift and splitting of the signals of the macrocycle hydroquinone protons f and g is indicative of aromatic donor–acceptor interactions between the electron-rich macrocycle hydroquinone units and the electron-poor pyridinium group. The downfield shift of the signals of the pyridine protons a and b is consistent with the withdrawal of electron density by the formation of the halogen bond. Finally, the upfield shift of pyridinium proton signal β is a result of catenane inter-ring halogen-bond formation between the pyridine nitrogen atom and the iodine substituent.

Two-dimensional ^1^H–^1^H ROESY spectroscopy was used to confirm the interlocked nature of the catenane. At 500 MHz, the spectrum in CDCl_3_ is broader than at 300 MHz, which is indicative of dynamic behavior on the NMR timescale. Heating to 70 °C in CD_3_CN resulted in sharper signals in the one-dimensional NMR spectrum, and through-space correlations between protons in the two ring components were identified in the ^1^H–^1^H ROESY spectrum (see Figure S3 in the Supporting Information).

The synthesis of the related protic and bromopyridinium functionalized catenanes was also attempted with threading components **4⋅BF_4_** and **5⋅BF_4_** by applying analogous RCM reaction conditions using a Grubbs catalyst. Although no evidence of catenane formation was observed with protic pyridinium thread **4⋅BF_4_**, with the bromopyridinium thread **5⋅BF_4_** a peak at *m*/*z*=1213.4 corresponding to the [2]catenane species was detected by electrospray mass spectrometry. In spite of numerous attempts, however, the catenane could not be isolated, which suggests it is produced in negligible yield.

To obtain further insights on the structure of catenane **7⋅BF_4_**, molecular dynamics (MD) simulations were performed in explicit chloroform by using the Amber12[11] accelerated GPU code.[12] The C=I⋅⋅⋅N halogen bond interaction was added to the general Amber force field (GAFF)[13] through a dummy atom,[14] which was parameterized as described in Supporting Information.

**Figure 4:**
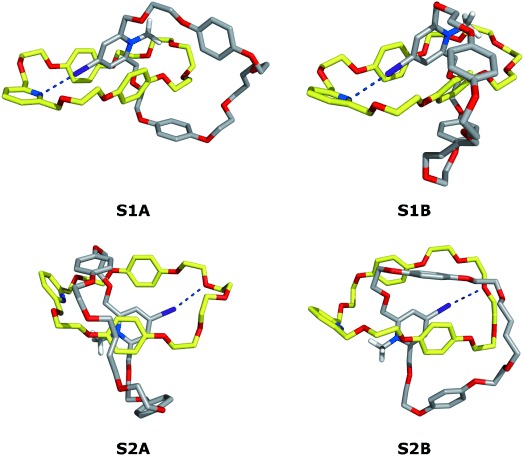
Starting co-conformations used in the MD simulations; S1A and S1B exhibit a C=I⋅⋅⋅N halogen bond and S2A and S2B present a C=I⋅⋅⋅O halogen bond. Macrocycle 1 is shown with yellow carbon atoms, and the iodopyridinium macrocycle is shown with gray carbon atoms. Hydrogen atoms apart those from the methyl groups were omitted for clarity. The C=I⋅⋅⋅N and C=I⋅⋅⋅O halogen bonds are drawn as blue dashed lines.

Four starting co-conformations were generated in the gas phase, as described in the Supporting Information and are presented in Figure 4.

These structures, **S1A**, **S1B**, **S2A**, and **S2B** correspond to two main arrangements: in **S1** (**A** and **B**) there is an I⋅⋅⋅N halogen bond as represented in Scheme 3, whereas in **S2** (**A** and **B**) co-conformations the iodopyridinium macrocycle is rotated with the C=I bond pointing towards the polyether loop of the pyridine macrocycle **1**. Within the two alternative binding scenarios, **A** and **B** differ mainly in the conformation of the iodopyridium macrocycle. Subsequently, these four co-conformations were subject to MD runs in chloroform for 50 ns.

**Figure 5:**
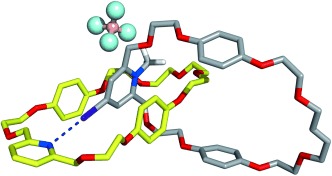
Representative co-conformation of 7⋅BF_4_ in chloroform solution for simulation of S1A. The BF_4_^−^ counteranion is represented in balls and sticks. The remaining details are similar to Figure 4.

The I⋅⋅⋅N halogen bond is highly stable throughout the course of the simulation time in both **S1A** and **S1B** co-conformations, as evident by the plot of the I⋅⋅⋅N distances versus the C=I⋅⋅⋅N angles presented in Figure S7 in the Supporting Information. A narrow spot centered at an I⋅⋅⋅N distance of 3.19 Å and a C=I⋅⋅⋅N angle of 173° is observed, which is indicative of the existence of a persistent halogen bond. A representative co-conformation of **7⋅BF_4_** in chloroform solution for simulation of **S1A** is presented in Figure 5. Simulation of **S1B** yields a virtually identical co-conformation (see Figure S8 in the Supporting Information). In this representative frame, as mentioned earlier, an I⋅⋅⋅N halogen bond is established between the iodopyridinium derivative and the pyridine ring of the macrocycle. Additionally, this co-conformation is stabilized by the stacking interactions between the iodopyridinium moiety and the hydroquinone rings, in total agreement with the NMR findings.

The alternative **S2** scenarios (**A** and **B**) allow the existence of C=I⋅⋅⋅O halogen bonds between the iodopyridinium and the oxygen atoms of the polyether loop of the pyridine macrocycle. Indeed, these noncovalent interactions were intermittently observed during the MD simulations undertaken with both binding arrangements. This type of halogen bond is more labile than the C=I⋅⋅⋅N ones, thereby leading to a diffuse distribution of the I⋅⋅⋅O distances and C=I⋅⋅⋅O angles when they are plotted together, as can be seen in Figure S9 in the Supporting Information, and therefore these halogen bonds are not sufficiently stable for XB templation. See the Supporting Information for a full discussion of the MD simulations carried out with **S2A** and **S2B**.

In conclusion, we designed a XB-acceptor pyridine-containing macrocycle and demonstrated the formation of pseudorotaxane assemblies with a series of XB-donor iodo-functionalized triazolium as well as bromo- and iodo-functionalized pyridinium threading components; the pseudorotaxane assemblies are stabilized by a charge-assisted XB interaction. The strength of the XB interpenetrative assembly between the pyridine macrocycle and iodopyridinium thread was exploited in the RCM clipping synthesis of a novel [2]catenane by using a Grubbs catalyst. The crucial importance of the single charge-assisted XB interaction between the two components was highlighted by the fact that no evidence of catenane formation was observed in an analogous RCM reaction of the pyridine macrocycle with the corresponding protic pyridinium reactant. Hence we have illustrated that XB has real potential in templating the construction of mechanically bonded molecular architectures.
