# Supplementary material for: A Catenane Assembled through a Single Charge-Assisted Halogen Bond
Source: Angew Chem Int Ed Engl. 2013 Mar 11;52(16):4356–60. doi: 10.1002/anie.201300464 (PMC4499249; doi:10.1002/anie.201300464)
Supplement: Supplementary file 1 [file anie0052-4356-sd1.pdf]

Angewandte Chemie
Eine Zeitschrift der Gesellschaft Deutscher Chemiker

Supporting Information

© Wiley-VCH 2013

69451 Weinheim, Germany

A Catenane Assembled through a Single Charge-Assisted Halogen Bond**

*Lydia C. Gilday, Thomas Lang, Antonio Caballero, Paulo J. Costa, Vítor Félix, and Paul D. Beer**

anie_201300464_sm_miscellaneous_information.pdf

SUPPORTING INFORMATION

TABLE OF CONTENTS

Part I: Experimental Procedures S2

General Notes S2

Synthesis S2

Pyridine macrocycle **1** S2

Halotriazolium thread components **2·BF$_4$**, **3·BF$_4$** S8

Halopyridinium thread components **4·BF$_4$**, **5·BF$_4$**, **6·BF$_4$** S15

Catenane **7·BF$_4$ S22**

Part II: Spectral Characterisation of Catenane 7·BF$_4$ S23

High resolution MS of catenane **7·BF$_4$** S23

^{13}C NMR spectrum of catenane **7·BF$_4$** S23

2D ^{1}H–^{1}H ROESY spectrum of catenane **7·BF$_4$** S24

Part III: Titration Protocols S24

Part IV: Molecular modelling S25

Part V: References S33

Part I: Experimental Procedures.

General Notes.

Commercially available solvents and chemicals were used without further purification unless stated. Where anhydrous solvents were used, they were degassed with nitrogen, dried by passing through a MBraun MPSP-800 column and then used immediately. Deionised water was used in all cases. Copper iodide, Tris[(1-Benzyl-1*H*-1,2,3-Triazol-4-yl)methyl]Amine (TBTA) and Grubbs' 2nd generation catalyst were stored in a desiccator. NMR spectra were recorded on Varian Mercury 300, Varian Unity Plus 500 and Bruker AVII 500 (with ^{13}C Cryoprobe) spectrometers. Mass spectra were carried out on Micromass LCT and Bruker microTOF spectrometers.

Syntheses.

Compounds **13**[1], **14**[2], **22**[3], **26**[4], **27**[5], **28**[6], and **29**[7] were prepared following literature procedures.

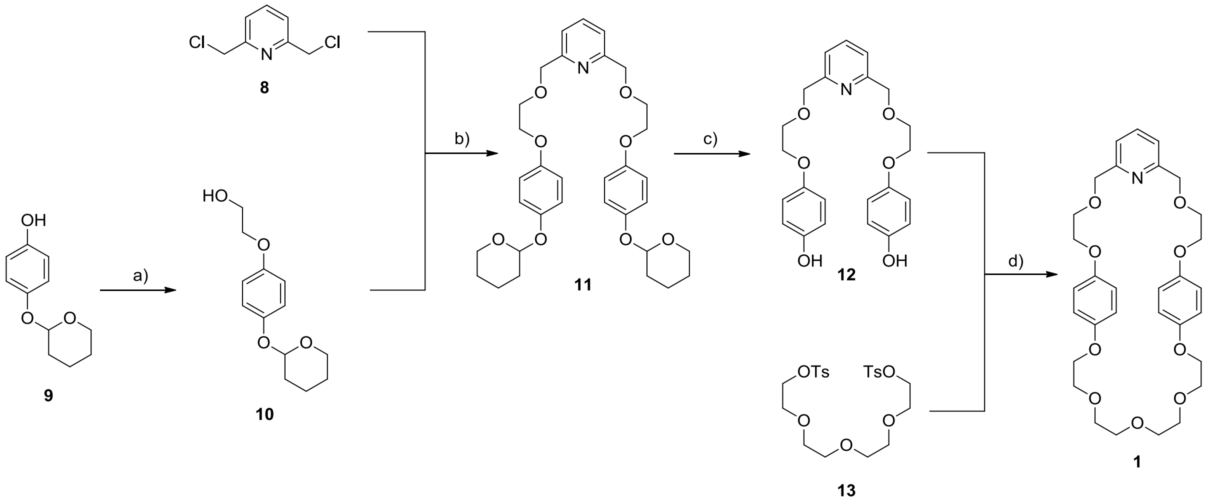

Scheme 1: Synthesis of pyridine macrocycle **1**. Reactions conditions: a) bromoethanol, K_2CO_3, DMF, 100 °C, 32%; b) NaH, THF, 45%; c) HCl, MeOH, 78%; d) Cs_2CO_3, DMF, 60 °C, 20%.

4-((tetrahydro-2H-pyran-2-yl)oxy)phenol 9:

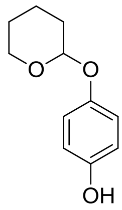

Hydroquinone (10.0 g, 91 mmol) and pyridinium *para*-toluenesulfonate (2.25 g, 9.1 mmol) were dissolved in degassed acetone (500 mL). A solution of 3,4-dihydro-2*H*-pyran (8.5 mL, 93 mmol) in degassed acetone (250 mL) was added dropwise and the reaction mixture stirred at r.t. for 6 days under N_2. The solvent was removed *in vacuo* and the crude brown oil was purified by column chromatography (Al_2O_3, EtOAc:cyclohexane 1:9 to 4:6) to yield the desired product **9** as a light yellow oil (5.22 g, 27 mmol, 29%).

^{1}H NMR (300 MHz, $CDCl_3$) δ (ppm): 6.93 (d, 2H, Ar, ^3J = 8.9 Hz, hydroquinone-Ar*H*), 6.72 (d, 2H, ^3J = 8.9 Hz, hydroquinone-Ar*H*), 5.28 (t, 1H, ^3J = 3.4 Hz, OC*H*), 4.98 (s, 1H, O*H*), 3.95 (m, 1H, OC*H*$_2$), 3.60 (m, 1H, OC*H*$_2$), 1.82–1.87 (2H, m, C*H*$_2$), 1.57–1.72 (m, 4H, C*H*$_2$); ESMS *m/z*: 193.1 ($[M-H]^-$, $C_{11}H_{14}O_3$ requires 193.1).

2-(4-((tetrahydro-2H-pyran-2-yl)oxy)phenoxy)ethanol 10:

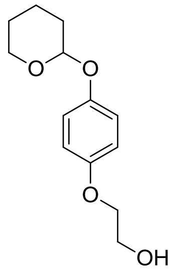

Compound **9** (5.22 g, 27 mmol) and K_2CO_3 (7.4 g, 54 mmol) were dissolved in anhydrous DMF (250 mL). A solution of bromoethanol (3.8 mL, 54 mmol) in anhydrous DMF (50 mL) was added dropwise. The reaction mixture was then heated at 100 °C for 24 hr under N_2. After cooling to r.t., the reaction mixture was filtered, and the solvent removed *in vacuo*. The residue was then redissolved in hot $CHCl_3$ (75 mL), filtered and solvent removed *in vacuo*.

The crude brown oil was purified by column chromatography (Al_2O_3, EtOAc:cyclohexane 1:4 to 1:0) to yield the desired product **10** as a light yellow oil (2.07 g, 8.7 mmol, 32%).

^{1}H NMR ($CDCl_3$, 300 MHz) δ (ppm): 6.99 (d, 2H, ^3J = 9.1 Hz, hydroquinone-*H*), 6.84 (d, 2H, ^3J = 9.1 Hz, hydroquinone-*H*), 5.30 (t, ^3J = 3.4 Hz, 1H, OC*H*), 4.03 (m, 2H, OCH_2), 3.93 (m, 3H, OCH_2), 3.59 (m, 1H, OCH_2), 1.82–1.87 (m, 2H, CH_2), 1.56–1.72 (m, 4H, CH_2); ^{13}C NMR ($CDCl_3$, 75.5 MHz) δ (ppm): 153.6, 151.6, 117.9, 115.5, 97.4, 69.9, 62.2, 61.7, 30.6, 25.4, 19.0; ESMS *m/z*: 261.1 ([M+Na]$^+$, $C_{13}H_{18}NaO$ requires 261.1).

THP-protected pyridine macrocycle precursor 11:

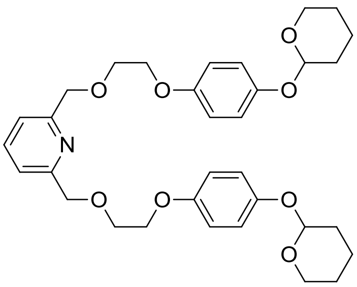

NaH (60% in oil, 348 mg, 8.7 mmol) was suspended anhydrous THF (25 mL) and cooled to 0 °C. A solution of compound **10** (2.07 g, 8.7 mmol) in anhydrous THF (25 mL) was added dropwise and the reaction mixture was stirred at 0 °C, under N_2 for 1 hr. A solution of 2,6-bis(chloromethyl)pyridine (695 mg, 3.9 mmol) in anhydrous THF (25 mL) was added dropwise at 0 °C and the reaction mixture was heated at reflux under N_2 for 5 days. After cooling to r.t., the solvent was removed *in vacuo*, the residue was redissolved in CH_2Cl_2 (75 mL) and washed with H_2O (2 x 40 mL) and brine (3 x 40 mL), dried over anhydrous $MgSO_4$, filtered and the solvent removed *in vacuo*. The crude residue was purified by column chromatography (Al_2O_3, EtOAc:cyclohexane 0:1 to 1:0) to yield the desired product **11** as a colourless oil (1.01 g, 1.8 mmol, 45%).

^{1}H NMR ($CDCl_3$, 300 MHz) δ (ppm): 7.70 (t, ^3J = 7.7 Hz, 1H, pyridine-H^4), 7.39 (d, ^3J = 7.7 Hz, 2H, pyridine-H^3&H^5), 6.98 (d, ^3J = 9.2 Hz, 4H, hydroquinone-*H*), 6.85 (d,

^{3}J = 9.2 Hz, 4H, hydroquinone-*H*), 5.29 (t, ^{3}J = 3.3 Hz, 2H, OC*H*), 4.73 (s, 4H, OCH_2), 4.13 (m, 4H, OCH_2), 3.90 (m, 6H, OCH_2), 3.58 (m, 2H, OCH_2), 1.81–1.85 (m, 4H, CH_2), 1.58–1.70 (m, 8H, CH_2); ^{13}C NMR ($CDCl_3$, 75.5 MHz) δ (ppm): 157.9, 153.8, 151.5, 137.5, 120.2, 117.9, 115.6, 97.4, 74.3, 69.6, 68.1, 62.2, 30.6, 25.4, 19.1; ESMS *m/z*: 580.2897 ($[M+H]^+$, $C_{33}H_{42}NO_8$ requires 580.2905).

Pyridine macrocycle precursor 12:

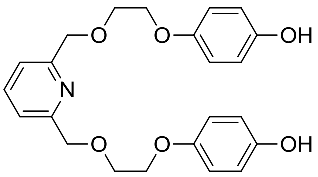

Compound **11** (2.01 g, 3.5 mmol) was dissolved in MeOH (200 mL), 600 μL of conc. $HCl_{(aq)}$ was added and the reaction mixture stirred at r.t. for 6 hr. The solution was neutralized with $NaHCO_{3(s)}$ and solvent was removed *in vacuo*. The solid was washed with Et_2O (10 x 20 mL) to yield the desired product as a light yellow oil (1.19 g, 2.9 mmol, 78%).

^{1}H NMR ($CDCl_3$, 300 MHz) δ (ppm): 7.73 (t, 1H, ^{3}J = 7.8 Hz, pyridine-H^4), 7.41 (d, ^{3}J = 7.8 Hz, 2H, pyridine-H^3&H^5), 6.73 (s, 8H, hydrquinone-*H*), 5.86 (br, 2H, O*H*), 4.73 (s, 4H, OCH_2), 4.05 (m, 4H, OCH_2), 3.87 (m, 4H, OCH_2); ^{13}C NMR ($CDCl_3$, 75.5 MHz) δ (ppm): 157.7, 152.8, 150.2, 145.5, 120.8, 116.2, 115.9, 73.8, 69.7, 68.1; ESMS *m/z*: 412.1742 ($[M+H]^+$, $C_{23}H_{26}NO_6$ requires 412.1755).

Pyridine macrocycle 1:

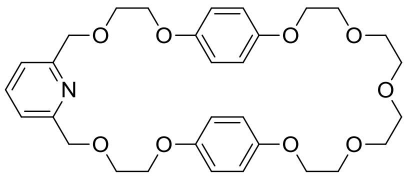

Cs_2CO_3 (1.66 g, 5.1 mmol) was suspended in anhydrous DMF (330 mL) and heated at 60 °C under N_2. Solutions of compound **12** (950 mg, 2.3 mmol) in anhydrous DMF (20 mL) and tetraethylene glycol bis-tosylate **13** (1.16 g, 2.3 mmol) in anhydrous DMF (20 mL) were added dropwise over a period of 20 hr. The reaction mixture was heated at 60 °C under N_2 for an additional 24 hr. After cooling to r.t., the reaction mixture was filtered and the solvent removed *in vacuo*. The residue was redissolved in hot $CHCl_3$ (40 mL), filtered and the solvent removed *in vacuo*. The crude brown oil was purified by column chromatography (SiO_2, EtOAc:cyclohexane 1:0 to 0:1) to yield the desired product **1** as a white solid. (260 mg, 0.46 mmol, 20%).

^{1}H NMR ($CDCl_3$, 300 MHz) δ (ppm): 7.60 (t, ^3J = 7.6 Hz, 1H, pyridine-H^4) 7.27 (d, ^3J = 7.6 Hz, 2H, pyridine-H^3&H^5), 6.76 (s, 8H, hydroquinone-*H*), 4.70 (s, 4H, OCH_2), 4.06 (m, 4H, OCH_2), 4.00 (m, 4H, OCH_2), 3.86 (m, 4H, OCH_2), 3.79 (m, 4H, OCH_2), 3.67 (m, 8H, OCH_2); ^{13}C NMR ($CDCl_3$, 75.5 MHz) δ (ppm): 157.8, 153.2, 153.0, 137.3, 120.8, 115.9, 115.7, 74.2, 70.9, 70.9(sic), 69.9, 69.4, 68.3, 68.2; ESMS *m/z*: 570.2699 ($[M+H]^+$, $C_{31}H_{40}NO_9$ requires 570.2698).

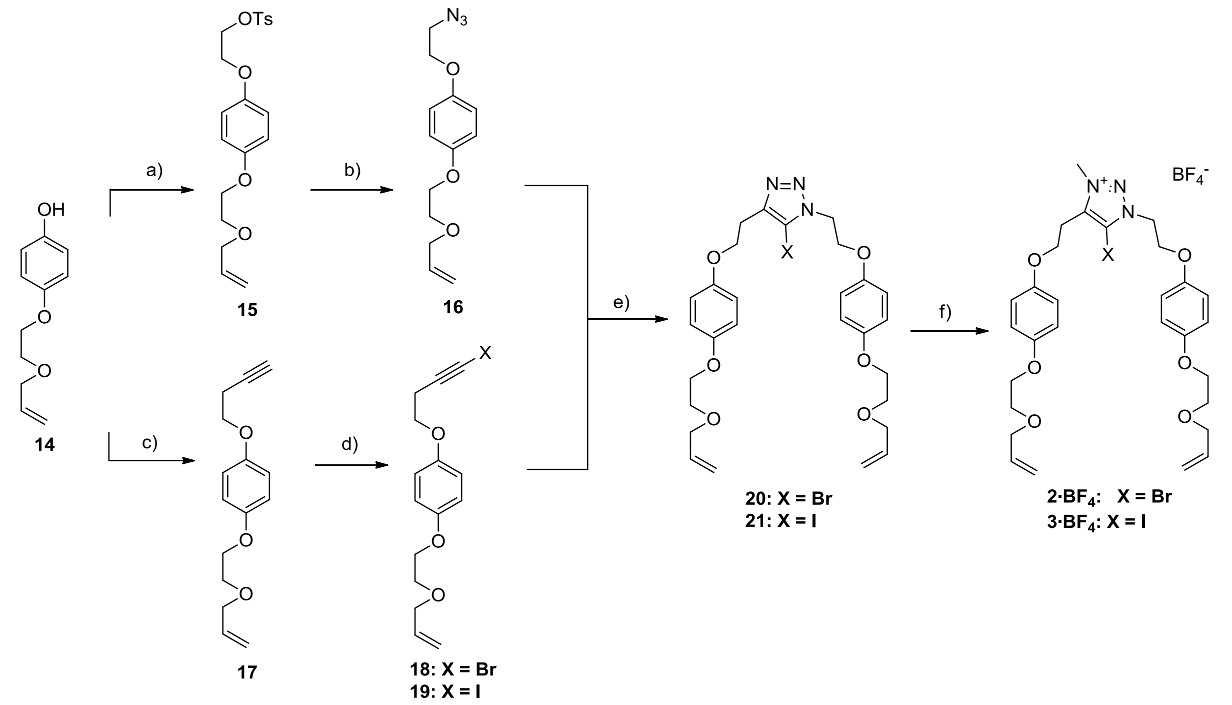

Scheme 2: Synthesis of halotriazolium threads **2·BF$_4$** and **3·BF$_4$**. Reactions conditions: a) K_2CO_3, ethyl-1,2-bis(4-methylbenzenesulfonate), MeCN, 45%; b) NaN_3, DMF, 85 °C, 98%; c) but-3-yn-1-ol, PPh_3. DEAD, THF, 45%; d) NBS or NIS, $AgNO_3$, acetone, 98%; e) **20**: CuBr, $Cu(AcO)_2$, THF, 38%; **21**: CuI, TBTA, 71%; f) Me_3OBF_4, CH_2Cl_2, **2·BF$_4$**: 87%, **3·BF$_4$**: 85%.

2-(4-(2-(allyloxy)ethoxy)phenoxy)ethyl-4-methylbenzenesulfonate, 15.

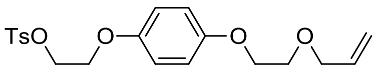

Compound 4-(2-(allyloxy)ethoxy)phenol **14** (0.5 g, 2.3 mmol) and K_2CO_3 (1.77 g, 12.8 mmol) were stirred for 20 min in MeCN (150 mL) and then ethyl-1,2-bis(4-methylbenzenesulfonate) (0.95 g, 2.6 mmol) was added and the reaction mixture heated to reflux under N_2 for 16 hr. After cooling to r.t. excess base was removed by filtration, the solvent removed *in vacuo* and then Et_2O was added to the crude residue precipitating the undesirable compounds. After filtering, the Et_2O was removed *in vacuo* giving the desired compound **15** as golden oil (0.41 g, 1.04 mmol, 45%).

^1H NMR (300 MHz, $CDCl_3$) δ (ppm): 7.80 (d, ^3J = 6 Hz, 2H, Ar*H*), 7.34 (d, ^3J = 6 Hz, 2H, Ar*H*), 6.81 (d, ^3J = 6 Hz, 2H, hydroquinone-*H*), 6.70 (d, ^3J = 6 Hz, 2H, hydroquinone-*H*), 5.87–5.98 (1H, m), 5.18–5.34 (m, 2H, C*H*$_2$), 4.31–4.34 (m, 2H, C*H*$_2$), 4.05–4.10 (6H, m),

3.75–3.78 (m, 2H, CH_2) 2.44 (s, 3H, CH_3); ^{13}C NMR (75.5 MHz, $CDCl_3$) δ (ppm): 153.5, 152.3, 134.6, 132.9, 129.8, 128.0, 117.4, 115.7, 115.6, 73.4, 68.6, 68.3, 68.0, 66.2, 21.7; ESMS *m/z*: 393.13 ($[M+H]^+$, $C_{20}H_{24}O_6S$ requires 393.13).

1-(2-(allyloxy)ethoxy)-4-(2-azidoethoxy)benzene, 16.

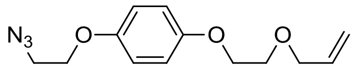

Compound **15** (0.41 g, 1.04 mmol) and NaN_3 (0.20 g, 3.07 mmol) in anhydrous DMF (20 mL) was stirred for 16 hr at 85 °C. The reaction was allowed to cool to r.t., poured into water (200 ml) at 0 °C and stirred for 10 min. The organic compounds were extracted with Et_2O (4 x 200 mL) and the combined organic extracts were dried over anhydrous $MgSO_4$, filtered, and the solvent removed *in vacuo* to give compound **16** as a golden oil (0.26 g, 0.99 mmol, 98%).

^{1}H NMR (300 MHz, $CDCl_3$) δ (ppm): 6.84 (d, ^3J = 6.0 Hz, 2H, hydroquinone-*H*), 6.81 (d, ^3J = 6 Hz, 2H, hydroquinone-Ar*H*), 5.87–5.94 (m, 1H, C=C*H*), 5.15–5.29 (m, 2H, C=CH_2), 4.31–4.34 (m, 2H, CH_2), 4.02–4.05 (m, 6H, CH_2), 3.73 (t, ^3J = 3 Hz, 2H, CH_2), 3.50 (t, ^3J = 3 Hz, 2H,CH_2); ^{13}C NMR (75.5 MHz, $CDCl_3$) δ (ppm): 152.5, 134.6, 117.4, 115.7, 115.6, 72.4, 68.6, 68.1, 67.6, 55.2; ESMS *m/z*: 264.13 ($[M+H]^+$, $C_{13}H_{18}N_3O_3$ requires 264.13).

1-(2-(allyloxy)ethoxy)-4-(but-3-ynyloxy)benzene, 17.

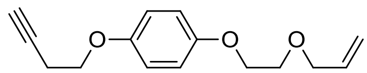

To a solution of 4-(2-(allyloxy)ethoxy)phenol **14** (1.09 g, 5.6 mmol), but-3-yn-1-ol (0.43 mL, 5.6 mmol) and PPh_3 (1.47 g, 56 mmol) in anhydrous THF (150 mL) at 0 °C was added dropwise 0.92 ml (5.6 mmol) of Diethyl azodicarboxylate (0.92 mL, 5.6 mmol). The resulting

solution was stirred at r.t. under N_2 for 16 hr. The solvent was removed *in vacuo* and the resultant residue was purified by column chromatography (SiO_2, 8:0.5 CH_2Cl_2:EtOH) to give the product **17** as a white solid (0.63 g, 2.56 mmol, 45%).

^{1}H NMR (300 MHz, $CDCl_3$) δ (ppm): 6.85 (s, 4H, hydroquinone-H), 5.87–6.00 (m, 1H, C=C*H*), 5.18–5.34 (m, 2H, C=C*H*$_2$), 4.06–4.10 (m, 4H, C*H*$_2$), 3.73 (t, ^3J = 3 Hz, 2H, C*H*$_2$), 3.77 (t, ^3J = 3 Hz, 2H, C*H*$_2$), 2.64 (2H, td, *J* = 9 Hz, *J* = 3Hz, C*H*$_2$), 2.03 (t, ^4J = 3 Hz, 1H alkyne-C*H*); ^{13}C NMR (75.5 MHz, $CDCl_3$) δ (ppm): 153.3, 152.7, 134.6, 117.4, 115.7, 115.6, 80.6, 72.4, 69.8, 68.6, 68.1, 66.7, 19.6; ESMS *m/z*: 247.13 ([M+H]$^+$, $C_{15}H_{19}O_3$ requires 247.13).

1-(2-(allyloxy)ethoxy)-4-(4-bromobut-3-ynyloxy)benzene 18

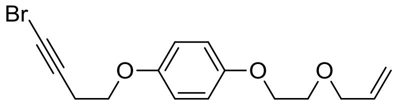

To a solution of compound **17** (0.26 g, 1 mmol) in acetone (20 mL) were added *N*-bromosuccinimide (0.23 g, 1.3 mmol) and $AgNO_3$ (18 mg, 0.1 mmol). The reaction mixture was left to stir for 16 hr at r.t. in the dark under N_2, after which a yellow suspension formed. All volatile components were removed *in vacuo* to give a yellow oil, which was purified by column chromatography (SiO_2, 98:2 CH_2Cl_2:MeOH) to give compound **17** as a white solid (0.32 g, 0.98 mmol, 98%);

^{1}H NMR (300 MHz, $CDCl_3$) δ (ppm): 6.87 (d, ^3J = 6 Hz, 2H, hydroquinone-*H*), 6.83 (d, ^3J = 6 Hz, 2H, hydroquinone-*H*), 5.88–6.01 (m, 1H, C=C*H*),5.19–5.34 (m, 2H, C=C*H*$_2$), 4.06–4.10 (m, 6H, C*H*$_2$), 3.78 (t, ^3J = 3 Hz, 2H), 3.66 (t, ^3J = 6 Hz, 2H, C*H*$_2$); ^{13}C NMR (75.5 MHz, $CDCl_3$) δ (ppm): 153.3, 152.6, 134.6, 117.4, 115.7, 115.6, 72.4, 68.6, 68.0, 66.4, 39.9, 20.8; ESMS *m/z*: 325.04 ([M+H]$^+$, $C_{15}H_{18}BrO_3$ requires 325.04).

1-(2-(allyloxy)ethoxy)-4-(4-iodobut-3-ynyloxy)benzene, 19.

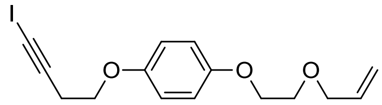

To a solution of compound **17** (0.28 g, 1.1 mmol) in acetone (20 mL) were added *N*-iodosuccinimide (0.31 g, 1.3 mmol) and $AgNO_3$ (23 mg, 0.13 mmol). The reaction mixture was left to stir for 16 hr at r.t. in the dark under $N_{2(g)}$, after which a yellow suspension formed. All volatile components were removed *in vacuo* to give a yellow oil, which was purified by column chromatography (SiO_2, 98:2 CH_2Cl_2:MeOH) to give compound **19** as a white solid (0.4 g, 1.07 mmol, 98%).

^{1}H NMR (300 MHz, $CDCl_3$) δ (ppm): 6.86 (d, ^3J = 6 Hz, 2H, hydroquinone-*H*), 6.81 (d, ^3J = 6 Hz, 2H, hydroquinone-H), 5.87–6.00 (m, 1H, C=C*H*), 5.21–5.33 (m, 2H, C=CH_2), 4.06–4.10 (m, 6H, CH_2), 3.77 (t, ^3J = 3 Hz, 2H, CH_2), 2.80 (t, ^3J = 6 Hz, 2H, CH_2); ^{13}C NMR (75.5 MHz, CD_2Cl_2) δ (ppm): 153.3, 152.6, 134.6, 117.4, 115.7, 115.6, 110.0, 90.49, 72.35, 68.6, 68.1, 66.6, 21.8; ESMS *m/z*: 373.02 ($[M+H]^+$, $C_{15}H_{18}IO_3$requires 373.02).

1,4-bis(2-(4-(2-(allyloxy)ethoxy)phenoxy)ethyl)-5-bromo-1H-1,2,3-triazole, 20.

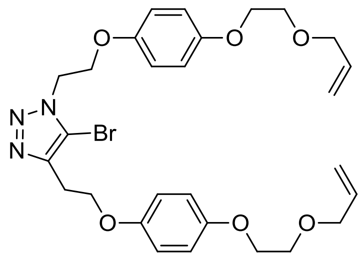

CuBr (0.14 g 0.98 mmol) and $Cu(AcO)_2$ (0.18 g, 0.98 mmol) were stirred in anhydrous THF (10 mL) for 20 min. This catalyst solution was added to a solution of bromoalkyne **18** (0.32 g, 0.98 mmol) and azide **16** (0.26 g, 0.98 mmol) and the reaction was stirred in the dark for 16 hr at 50 °C before being quenched with 10% $NH_4OH_{(aq)}$ solution (2 mL). Volatile components were removed *in vacuo*, H_2O (20 mL) was added and the aqueous phase was

extracted with CH_2Cl_2 (3 x 20 mL) The combined organic phases were dried over anhydrous $MgSO_4$, filtered and concentrated to dryness on a rotary evaporator to give a pale yellow solid. The crude product was purified by column chromatography (SiO_2, 95:5 CH_2Cl_2:MeOH) to give compound **20** as a white solid (0.22 g, 25.9 mmol, 38%).

^{1}H NMR (300 MHz, $CDCl_3$) δ (ppm): 6.74–6.86 (m, 8H, hydroquinone-*H*), 5.87–5.98 (m, 2H, C=C*H*), 5.18–5.33 (m, 4H, C=CH_2), 4.69 (t, ^3J = 6 Hz, 2H, CH_2), 4.35 (t, ^3J = 6 Hz, 2H, CH_2), 4.21 (t, ^3J = 6 Hz, 2H, CH_2), 4.09–4.04 (m, 8H, CH_2), 3.76 (t, ^3J = 3 Hz, 4H, CH_2), 3.12 (t, ^3J = 6 Hz, 2H, CH_2); ^{13}C NMR (75.5 MHz, $CDCl_3$) δ (ppm): 153.6, 153.1, 153.0, 152.14, 143.24, 134.5, 117.33, 115.6, 115.5, 72.4, 68.6, 68.1, 66.7, 66.6, 48.4, 25.7; ESMS *m/z*: 588.16 ($[M+H]^+$, $C_{28}H_{34}BrN_3O_6$ requires 588.16).

1,4-bis(2-(4-(2-(allyloxy)ethoxy)phenoxy)ethyl)-5-iodo-1H-1,2,3-triazole, 21.

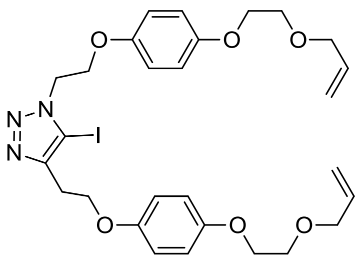

CuI (0.063 g 0.33 mmol) and TBTA (0.17 g, 0.33 mmol) were stirred in anhydrous THF (10 ml) for 20 min. The catalyst solution was added to a solution of iodoalkyne **19** (0.31 g, 0.83 mmol) and azide **16** (0.22 g, 0.83 mmol) and the reaction left to stir in the dark for 16 hr at r.t. before being quenched with 10% $NH_4OH_{(aq)}$ solution (2 mL). Volatile components were removed *in vacuo*. H_2O (20 mL) was added and the aqueous phase was extracted with CH_2Cl_2 (3 x 20 mL). The combined organic fractions were dried over anhydrous $MgSO_4$, filtered and concentrated to dryness on a rotary evaporator to give a pale yellow solid. The crude product was purified by column chromatography (SiO_2, 95:5 CH_2Cl_2:MeOH) to give yellow oil (0.150 g, 0.24 mmol, 71 %).

^{1}H NMR (300 MHz, $CDCl_3$) δ (ppm): 6.76–6.76 (m, 8H, hydroquinone-*H*), 5.88–5.99 (m, 2H, C=C*H*), 5.20–5.34 (m, 4H, C=CH_2), 4.74 (t, ^{3}J = 6 Hz, 2H, CH_2), 4.37 (t, ^{3}J = 6 Hz, 2H, CH_2), 4.22 (t, ^{3}J = 6 Hz, 2H, CH_2), 4.05–4.10 (m, 8H, CH_2), 3.78 (t, ^{3}J = 3 Hz, 4H, CH_2), 3.15 (2H, t, ^{3}J = 6 Hz); ^{13}C NMR (75.5 MHz, $CDCl_3$) δ 153.7, 153.3, 153.0, 152.4, 143.2, 134.5, 117.4, 115.6, 115.5, 72.6, 68.9, 68.3, 66.7, 66.7, 48.5, 25.8; ESMS *m/z*: 636.15 ($[M+H]^+$, $C_{28}H_{34}IN_3O_6$ requires 636.15).

1,4-bis(2-(4-(2-(allyloxy)ethoxy)phenoxy)ethyl)-5-bromo-1H-1,2,3-triazol-3-ium tetrafluoroborate, 2·BF_4.

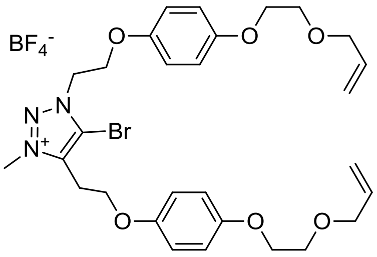

Bromotriazole **20** (0.22 g, 0.37 mmol) was dissolved in anhydrous CH_2Cl_2 (25 mL), Me_3OBF_4 (0.082 g, 0.55 mmol) was added, and the reaction mixture stirred at r.t. under N_2 for 48 hr. MeOH (2 mL) was added and all volatile components were removed *in vacuo* to give a yellow oil, which was purified by column chromatography (SiO_2, 98:2 CH_2Cl_2:MeOH) to give a yellow oil (0.22 g, 0.32 mmol, 87%).

^{1}H NMR (300 MHz, $CDCl_3$) δ (ppm): 6.72–6.82 (m, 8H, hydroquinone-*H*), 5.87–5.98 (m, 2H, C=C*H*), 5.18–5.32 (m, 4H, C=CH_2), 4.90 (t, ^{3}J = 6 Hz, 2H, CH_2), 4.46 (t, ^{3}J = 6 Hz, 2H, CH_2), 4.37 (s, 3H, CH_3), 4.18 (t, ^{3}J = 6 Hz, 2H, CH_2), 4.03–4.07 (m, 8H, CH_2), 4.05 (t, ^{3}J = 3Hz, 4H, CH_2), 3.76 (t, ^{3}J = 6 Hz, 2H, CH_2); ^{13}C NMR (75.5 MHz, CD_2Cl_2) δ 153.4, 153.5, 151.9, 151.6, 146.1, 134.6, 117.4, 115.7, 115.6, 115.5, 115.3, 89.5, 71.9, 68.4, 65.5, 65.4, 58.7, 53.8, 39.1, 25.4; ESMS *m/z*: 602.19, ($[M-BF_4]^+$, $C_{29}H_{37}BrN_3O_6$ requires 602.19).

1,4-bis(2-(4-(2-(allyloxy)ethoxy)phenoxy)ethyl)-5-iodo-1H-1,2,3-triazol-3-ium tetrafluoroborate, 3·BF_4.

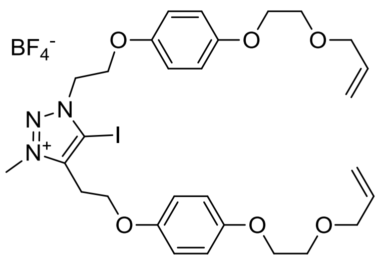

Iodotriazole **21** (0.250 g, 0.39 mmol) was dissolved in anhydrous CH_2Cl_2 (25 mL), Me_3OBF_4 was added (0.145 g, 0.97 mmol) and the reaction mixture stirred at r.t., under N_2 for 48 hr. MeOH (2 mL) was added and all volatile components were removed *in vacuo* to give a yellow oil, which was purified by column chromatography (SiO_2, 98:2 CH_2Cl_2:MeOH) to give a yellow oil (0.24 g, 0.33 mmol, 85%).

^{1}H NMR (300 MHz, $CDCl_3$) δ (ppm): 6.67–6.77 (m, 8H, hydroquinone-*H*), 5.82–5.92 (m, 2H, C=C*H*), 5.13–5.29 (m, 4H, C=CH_2), 4.82 (t, ^3J = 6 Hz, 2H, CH_2), 4.37 (t, ^3J = 6 Hz, 2H, CH_2), 4.31 (s, 3H, CH_3), 4.11 (t, ^3J = 6Hz, 2H, CH_2), 3.96–4.03 (m, 8H, CH_2), 3.70 (t, ^3J = 3 Hz, 4H, CH_2), 3.30 (t, ^3J = 6Hz, 2H, CH_2); ^{13}C NMR (75.5 MHz, CD_2Cl_2) δ (ppm): 153.7, 153.6, 151.8, 151.6, 146.0, 134.5, 117.4, 115.7, 115.6, 115.5, 115.3, 89.9, 72.3, 68.5, 65.4, 65.2, 58.3, 53.8, 39.1, 25.4; ESMS *m/z*: 650.17 ([M−BF_4]$^+$, $C_{29}H_{37}IN_3O_6$ requires 650.17).

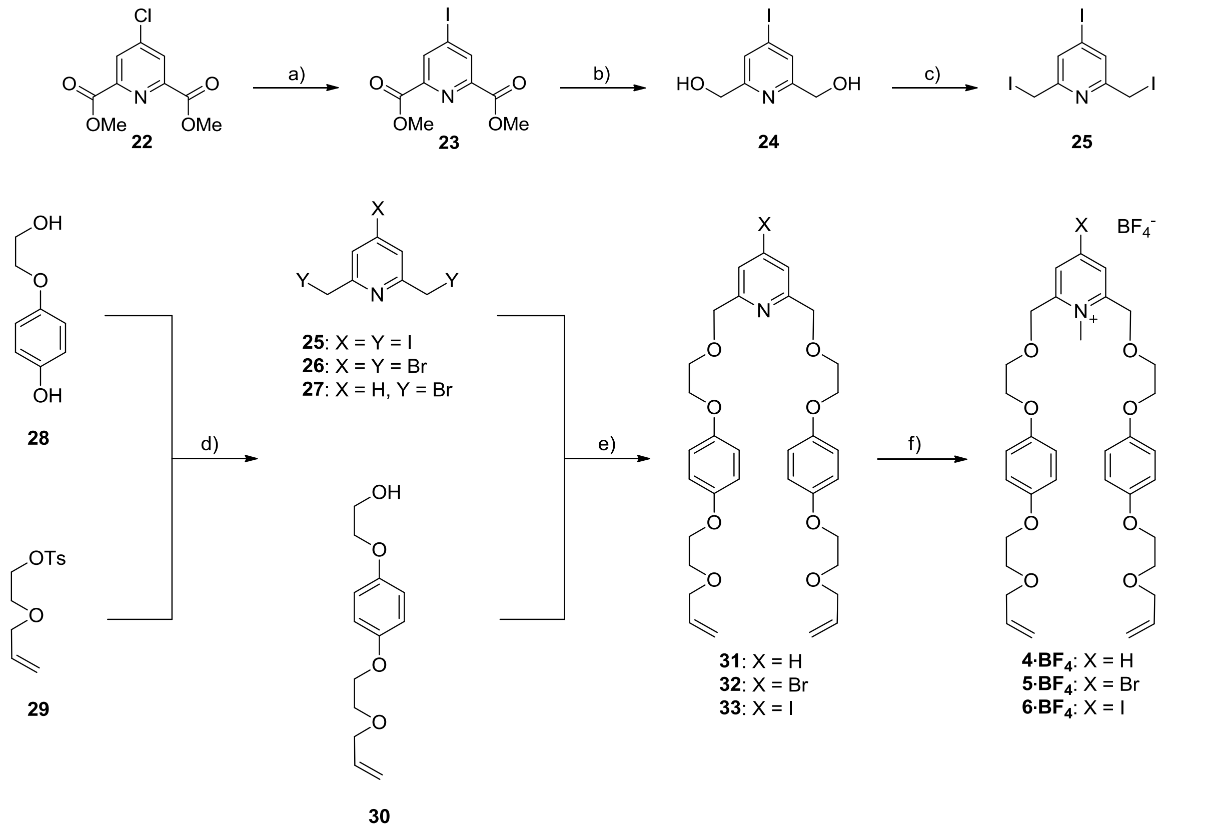

Scheme 3: Synthesis of halopyridinium threads **4·BF$_4$**, **5·BF$_4$** and **6·BF$_4$** Reaction conditions: a) NaI, acetyl chloride, MeCN, 74%;b) $NaBH_4$, MeOH, 68%; c) imidazole, PPh_3, I_2, CH_2Cl_2, 32%; d) Na^{t}BuO, MeCN, 81%, e) NaH, THF, **31**: 82%, **32**: 85%, **33**: 56%; f) Me_3BF_4, CH_2Cl_2, **4·BF$_4$**:33%; **5·BF$_4$**:38%; **6·BF$_4$**: 51%.

Dimethyl 4-iodopyridine-2,6-dicarboxylate, 23.

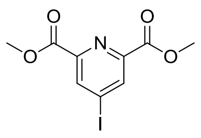

Compound **23** (2.00 g, 8.73 mmol) was dissolved in MeCN (100 mL), NaI (13.1 g, 87.3 mmol) was added and the reaction mixture was sonicated (bath sonicator) for 30 min. Acetyl chloride (1.86 mL, 26.2 mmol) was added and the reaction mixture sonicated for a further 45 min. CH_2Cl_2 (100 mL) and sat. $Na_2CO_{3(aq)}$ (100 mL) were added, the organic phase was separated and the aqueous phase extracted with CH_2Cl_2 (3 x 100 mL). The combined organic phases were washed with sat. $Na_2S_2O_3{\cdot}5H_2O_{(aq)}$ (1 x 200 mL), H_2O (1 x 200 mL), dried over anhydrous $MgSO_4$, filtered and the solvent removed *in vacuo* to give **24** as a white solid in 74% yield (2.06 g, 6.42 mmol).

^1H NMR (300 MHz, $CDCl_3$) δ (ppm): 8.67 (s, 2H, pyridine-H^3&H^5), 4.03 (s, 6H, CH_3); ESMS *m/z*: 344.0 ($[M+Na]^+$, $C_9H_8INNaO_4$ requires 343.9).

(4-Iodopyridine-2,6-diyl)dimethanol, 24.

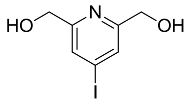

Compound **23** (750 mg, 2.34 mmol) was dissolved in MeOH (50 mL) and cooled to 0 °C. $NaBH_4$ (266 mg, 7.02 mmol) was added and the reaction mixture heated at reflux for 16 hr. After cooling to r.t. acetone (20 mL) was added and the solvent removed *in vacuo*. Sat. $NaHCO_{3(aq)}$ (3.5 mL) was added and the suspension heated to boiling forming a yellow solution. H_2O (4.5 mL) was added and the resulting precipitate cooled at 4 °C for 16 hr. The mixture was filtered and the solid dried in air to give compound **24** as a white solid (419 mg, 1.58 mmol, 68%).

^1H NMR (300 MHz, acetone-d_6) δ (ppm): 7.78 (s, 2H, pyridine-H^3&H^5), 4.63 (s, 4H, CH_2), 4.53 (s, 2H, OH); ESMS *m/z*: 266.0 ($[M+H]^+$, $C_7H_9INO_2$ requires 266.0).

4-iodo-2,6-bis(iodomethyl)pyridine, 25.

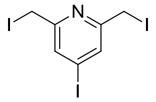

Compound **24** (300 mg, 1.13 mmol), imidazole (720 mg, 2.74 mmol) and PPh_3 (633 mg, 9.30 mmol) were dissolved in anhydrous CH_2Cl_2 (100 mL). A solution of I_2 (723 mg, 2.85 mmol) in anhydrous CH_2Cl_2 (100 mL) was added in portions and the reaction stirred at r.t. under N_2 for 16 hr. The solvent was removed *in vacuo* and the crude residue purified by column chromatography (SiO_2, CH_2Cl_2) to give **30** as an orange solid (174 mg, 0.36 mmol, 32%).

^1H NMR (300 MHz, C_6D_6) δ (ppm): 6.83 (s, 2H, pyridine-H^3&H^5), 3.72 (s, 4H, CH_2). ESMS *m/z*: 485.7710 ($[M+H]^+$, $C_7H_7I_3N$ requires 485.7707). This compound was used immediately without further characterisation.

2-(4-(2-(Allyloxy)ethoxy)phenoxy)ethanol, 30.

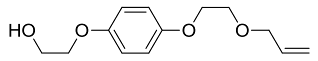

Compound **28** (1.80 g, 11.7 mmol), compound **29** (3.00 g, 11.7 mmol) and sodium *tert*-butoxide (1.27 g, 13.2 mmol) were dissolved in MeCN (150 mL) and heated at reflux for 48 hr under N_2. The mixture was cooled and filtered through Celite®. The solvent was removed *in vacuo* and the resulting residue was purified by column chromatography to give compound **32** as a cream coloured solid (2.26 g, 9.48 mmol, 81%).

^1H NMR (300 MHz, $CDCl_3$) δ (ppm): 6.83–6.90 (m, 4H, hydroquinone-*H*), 5.89–6.02 (m, 1H, C=C*H*), 5.19–5.35 (m, 2H, C=CH_2), 4.08–4.12 (m, 4H, CH_2), 4.03–4.06, (m, 2H, CH_2), 3.93–3.97 (m, 2H, CH_2), 3.79 (t, ^3J = 5.0 Hz, 2H, CH_2), 2.02 (br s, 1H, O*H*); ^{13}C NMR (75.5 MHz, $CDCl_3$) δ (ppm):153.3, 152.9, 134.5, 117.4, 115.6, 115.4, 72.4, 69.8, 68.6, 68.0, 61.6; ESMS *m/z*: 261.1097 ($[M+Na]^+$, $C_{13}H_{18}O_4$ requires 261.1107).

2,6-bis((2-(4-(2-(allyloxy)ethoxy)phenoxy)ethoxy)methyl)pyridine, 31.

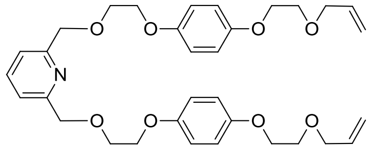

NaH (60% dispersion in mineral oil, 168 mg, 4.20 mmol) was suspended in anhydrous THF (50 mL), cooled to 0 °C and compound **30** (224 mg, 0.85 mmol) in anhydrous THF (10 mL) was added dropwise. After stirring at r.t. under N_2 for 30 min compound **27** was added. The reaction mixture was stirred at r.t. under N_2 for 2 weeks after which H_2O (100 mL) was added and the THF removed *in vacuo*. The aqueous mixture was extracted with CH_2Cl_2

(3 x 100 mL), dried over anhydrous $MgSO_4$, filtered and the solvent removed *in vacuo*. The crude residue was purified by column chromatography (SiO_2, 9:1 CH_2Cl_2:acetone) to give **31** as a white solid (406 mg, 0.70 mmol, 82%).

^{1}H NMR (300 MHz, $CDCl_3$) δ (ppm): 7.70 (t, ^3J = 8.1 Hz, 1H, pyridine-H^4), 7.40 (d, ^3J = 8.1 Hz, 2H, pyridine-H^3&H^5), 6.86 (s, 8H, hydroquinone-*H*), 5.88–6.01 (m, 2H, C=C*H*), 5.18–5.34 (m, 4H, C=CH_2), 4.73 (s, 4H, CH_2O), 4.06–4.15 (m, 12H, CH_2), 3.90 (t, ^3J = 4.8 Hz, 4H, CH_2), 3.77 (t, ^3J = 5.0 Hz, 4H, CH_2); ^{13}C NMR (75.5 MHz, $CDCl_3$) δ (ppm): 157.7, 153.0, 137.2, 134.5, 120.0, 117.2, 115.5, 74.0, 72.3, 69.4, 68.5, 67.9, 67.9 (*sic*), 30.2, 29.6. ESMS *m/z*: 580.2891 ($[M+H]^+$, $C_{33}H_{42}NO_8$ requires 580.2905).

2,6-bis((2-(4-(2-(allyloxy)ethoxy)phenoxy)ethoxy)methyl)-4-bromopyridine, 32.

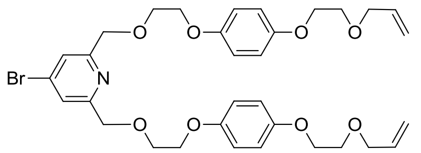

NaH (60% dispersion in mineral oil, 270 mg, 6.75 mmol) was suspended in anhydrous THF (50 mL) and cooled to 0 °C under N_2.Copmound **30** (523 mg, 2.19 mmol) was dissolved in anhydrous THF (20 mL) and added dropwise to the first solution. The mixture was stirred for at r.t. under N_2 for 30 min. Compound **26** (301 mg, 0.88 mmol) was added and the reaction stirred at r.t. under N_2 for 6 days. H_2O (50 mL) was added, the volatiles removed *in vacuo* and the aqueous phase was extracted with CH_2Cl_2 (3 x 100 mL). The combined organic phases were dried over anhydrous $MgSO_4$, filtered and the solvent removed *in vacuo*. The crude residue was purified using column chromatography (SiO_2, 9:1 $CHCl_3$:acetone) to give **32** as a creamy solid (487 mg 0.73 mmol, 85%).

^{1}H NMR (300 MHz, $CDCl_3$) δ (ppm): 7.61 (s, 2H, pyridine-H^3&H^5), 6.88 (s, 8H, hydroquinone-*H*), 5.89–6.02 (m, 2H, C=C*H*), 5.19–5.35 (m, 4H, C=CH_2), 4.70 (s, 4H, CH_2O), 4.15 (t, ^3J = 4.5 Hz, 4H, CH_2), 4.08–4.12 (m, 8H, CH_2), 3.91 (t, ^3J = 5.0 Hz, 4H,

CH_2), 3.79 (t, ^3J = 4.5 Hz, 4H, CH_2); ^{13}C NMR (75.5 MHz, $CDCl_3$): 159.4, 153.2, 153.0, 134.5, 123.2, 117.3, 115.6, 115.5, 73.4, 72.4, 69.7, 68.6, 68.0, 67.9; ESMS *m/z*: 680.1845 ($[M+Na]^+$, $C_{33}H_{40}BrNaNO_8$ requires 680.1830).

2,6-bis((2-(4-(2-(allyloxy)ethoxy)phenoxy)ethoxy)methyl)-4-iodopyridine, 33.

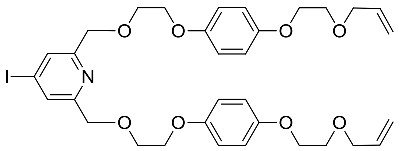

NaH (107 mg, 2.68 mmol) was suspended in anhydrous THF (100 mL) and cooled to 0 °C. **30** (250 mg, 1.05 mmol) was added and stirred at r.t. under N_2 for 30 min. Compound **25** (174 mg, 0.36 mmol) was added and the reaction mixture stirred at r.t. under N_2 for 72 hr. H_2O (100 mL) was added and the THF removed *in vacuo*. The aqueous mixture was extracted with CH_2Cl_2 (3 x 100 mL) and the combined organic extracts dried over anhydrous $MgSO_4$, filtered and the solvent removed *in vacuo*. The crude residue was purified by column chromatography (SiO_2, 9:1 CH_2Cl_2:acetone) to give **33** as a pale yellow solid (144 mg, 0.20 mmol, 56%).

^{1}H NMR (300 MHz, $CDCl_3$) δ (ppm): 7.82 (s, 2H, pyridine-H^3&H^5), 6.88 (s, 8H, hydroquinone-*H*), 5.89–6.02 (m, 2H, C=C*H*), 5.19–5.35 (m, 4H, C=CH_2), 4.68 (s, 4H, OCH_2) 4.15 (t, ^3J = 4.7 Hz, 4H, CH_2), 4.08–4.11 (m, 8H, CH_2), 3.91 (t, ^3J = 4.7 Hz, 4H, CH_2), 3.79 (t, ^3J = 5.0 Hz, 4H, CH_2); ^{13}C NMR (75.5 MHz, $CDCl_3$) δ (ppm): 152.7, 152.5, 146.5, 134.1, 128.7, 116.9, 115.15, 115.07, 103.1, 72.8, 71.9, 69.3, 68.2, 67.6, 67.4; ESMS *m/z*: 728.1699 ($[M+Na]^+$, $C_{33}H_{40}INNaO_8$ requires 728.1691).

Pyridinium tetrafluoroborate thread, 4·BF$_4$.

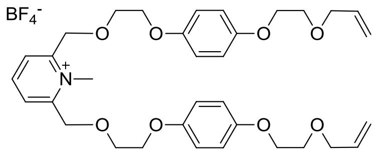

Compound **31** (100 mg, 0.17 mmol) was dissolved in anhydrous CH_2Cl_2 (50 mL) and Me_3OBF_4 (30.6 mg, 0.21 mmol) was added. The reaction mixture was stirred at r.t. under N_2 for 72 hr after which, MeOH (1 mL) was added and the solvent removed *in vacuo*. The crude residue was purified by column chromatography (SiO_2, 95:5 CH_2Cl_2:MeOH) to give **4·BF$_4$** as a pale yellow oil (33.2 mg, 55.8 μmol, 33%).

^{1}H NMR (300 MHz, $CDCl_3$) δ (ppm): 8.29 (t, ^3J = 8.0 Hz, 1H, pyridinium-H^4), 8.03 (d, ^3J = 8.0 Hz, 2H, pyridinium-H^3&H^5), 6.80-6.87 (m, 8H, hydroquinone-*H*), 5.88–6.01 (m, 2H, C=C*H*), 5.19–5.35 (m, 4H, C=CH_2), 5.00 (s, 4H, CH_2), 4.06–4.14 (m, 15H, CH_2 & NCH_3), 4.02–3.99 (m, 4H, CH_2), 3.78 (t, ^3J = 4.7 Hz, 4H, CH_2); ^{13}C NMR (75.5 MHz, $CDCl_3$) δ (ppm): 155.5, 153.3, 152.6, 144.7, 134.5, 126.1, 117.3, 115.6, 115.5, 72.3, 70.5, 68.6, 68.0, 67.6, 39.2. ^{19}F NMR (282.5 MHz, $CDCl_3$) δ (ppm): −153.1–−153.0 (m, BF_4). ESMS *m/z*: 594.3047 ($[M-BF_4]^+$, $C_{34}H_{44}NO_8$ requires 594.3061).

4-Bromopyridinium tetrafluoroborate thread, 5·BF$_4$.

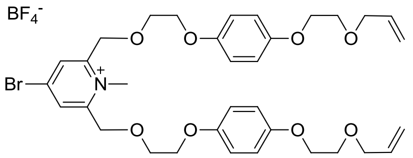

Compound **32** (50 mg, 0.076 mmol) was dissolved in anhydrous CH_2Cl_2 (30 mL). Me_3OBF_4 (15 mg, 0.1 mmol) was added and the reaction stirred at r.t. under N_2 for 4 days. MeOH (1 mL) was added and the solvent removed *in vacuo*. The crude residue was purified using preparative thin layer chromatography (SiO_2, 9:1 CH_2Cl_2:MeOH) to yield **5·BF$_4$** as a pale orange oil (22 mg, 0.030 mmol, 38%).

^{1}H NMR (300 MHz, $CDCl_3$) δ (ppm): 8.22 (s, 2H, pyridinium-H^3&H^5), 6.85 (s, 8H, hydroquinone-*H*), 5.88–6.01 (m, 2H, C=C*H*), 5.19–5.34 (m, 4H, C=CH_2), 4.94 (s, 4H, OCH_2), 4.00–4.14 (m, 16H, CH_2), 3.97 (s, 3H, CH_3), 3.77 (t, 4H, ^3J = 5.0 Hz, CH_2); ^{13}C NMR (75.5 MHz, $CDCl_3$) δ (ppm): 153.3, 152.6, 143.6, 134.5, 128.7, 117.3, 115.6, 115.4, 72.3, 70.7, 68.6, 68.0, 67.9, 38.8; ^{19}F NMR (282.5 MHz, $CDCl_3$) δ (ppm): −153.1–−153.2 (m, BF_4). ESMS *m/z*: 672.2163 ($[M-BF_4]^+$, $C_{34}H_{43}BrNO_8$ requires 672.2167).

4-Iodopyridinium tetrafluoroborate thread, 6·BF_4.

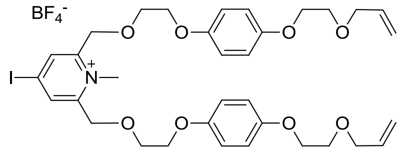

Compound **33** (28 mg, 37.9 μmol) was dissolved in anhydrous CH_2Cl_2 (10 mL). Me_3OBF_4 (7.60 mg, 51.4 μmol) was added and the reaction mixture was stirred at r.t. under N_2 for 48 hr. MeOH (1 mL) was added and the solvent removed *in vacuo*. The crude residue was purified by preparative thin layer column chromatography (SiO_2, 95:5 CH_2Cl_2:CH_3OH) to give **6·BF_4** as a pale orange oily solid (15.5 mg, 19.4 μmol, 51%).

^{1}H NMR (300 MHz, $CDCl_3$) δ (ppm): 8.41 (2H, s, pyridinium-H^3&H^5), 6.86 (s, 8H, hydroquinone-*H*), 5.88–6.01 (m, 2H, C=C*H*), 5.20–5.35 (m, 4H, C=CH_2), 4.13–4.15 (m, 4H, CH_2), 4.07–4.10 (m, 8H, CH_2), 4.01–4.04 (m, 4H, CH_2), 3.98 (s, 3H, CH_3), 3.78 (t, ^3J = 4.9 Hz, 4H, CH_2). ^{13}C NMR (75.5 MHz, $CDCl_3$) δ (ppm): 154.8, 153.3, 152.7, 135.0, 134.5, 117.4, 115.7, 115.5, 72.3, 70.7, 68.6, 68.0, 67.7, 67.5, 38.9; ^{19}F NMR (282.5 MHz, $CDCl_3$) δ (ppm): −153.1–−153.0 (m, BF_4). ESMS *m/z*: 720.2018 ($[M-BF_4]^+$, $C_{34}H_{43}INO_8$ requires 720.2028).

Catenane 7·BF$_4$

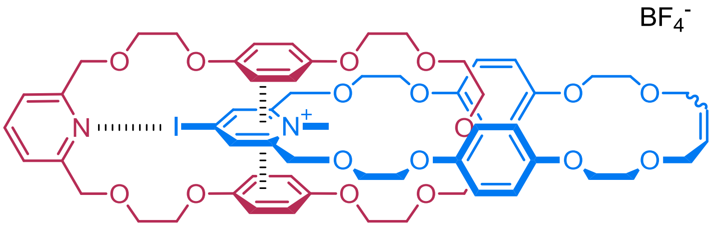

Compound **6·BF$_4$** (75.6 mg, 97.0 µmol) and pyridine macrocycle **1** (50 mg, 88.0 µmol) were dissolved in anhydrous CH_2Cl_2 (3 mL). Grubbs' II catalyst (10% by wt, 7.6 mg) was added and the reaction mixture stirred at r.t. under N_2 for 4 days. A further portion of Grubbs' II catalyst (3.5 mg) was added and the reaction mixture stirred for a further 24 hr. The solvent was removed *in vacuo* and the crude residue purified by successive preparative thin layer chromatography plates (SiO_2, 9:1 CH_2Cl_2:CH_3OH, 9:1 EtOAc:CH_3CN and 1:1 CH_2Cl_2:CH_3CN) to give **7·BF$_4$** as a white solid (8.56 mg, 6.35 µmol, 6.5%).

^{1}H NMR (300 MHz, $CDCl_3$) δ (ppm): 7.79 (t, ^3J = 7.6 Hz, 1H, pyridine-H^4), 7.38–7.40 (m, 4H, pyridine-H^3&H^5, pyridinium-H^3&H^5), 6.84–6.87 (m, 4H, hydroquinone-*H*), 6.77–6.80 (m, 4H, hydroquinone-*H*), 6.43–6.46 (m, 4H, hydroquinone-*H*), 6.31–6.34 (m, 4H, hydroquinone-*H*), 5.79 (s, 2H, alkene-C*H*), 4.89 (s, 4H, $^+N(CH_3)CCH_2$), 4.19–4.22 (m, 4H, CH_2), 4.11–4.12 (m, 4H, CH_2), 3.97–4.04 (m, 15H, CH_2, CH_3), 3.93–3.95 (s, 4H, CH_2), 3.72–3.75 (m, 4H, CH_2), 3.57–3.65 (m, 12H, CH_2), 3.46 (br s, 4H, CH_2), ^{13}C NMR (125.8 MHz, $CDCl_3$) δ (ppm): 153.1, 152.80, 152.75, 152.7, 152.3, 134.4, 128.9, 115.6, 115.4, 115.3, 115.0, 71.1, 70.5, 70.0, 69.9, 69.6, 69.0, 68.2, 68.1, 67.6, 67.1, 66.7, 29.7, 14.1; ^{19}F NMR (282.5 MHz, acetone-d$_6$) δ (ppm): −151.83–−151.78 (m, BF_4); ESMS *m/z*: 1261.4345 ($[M-BF_4]^+$, $C_{63}H_{78}IN_2O_{17}$ requires 1261.4340).

Part II: Spectral Characterisation of Catenane 7·BF_4.

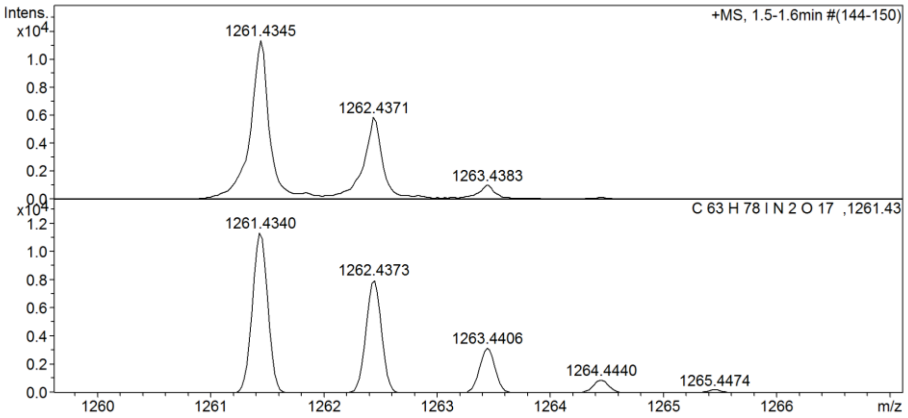

Figure S1: High resolution mass spectrum of catenane **7·BF_4**

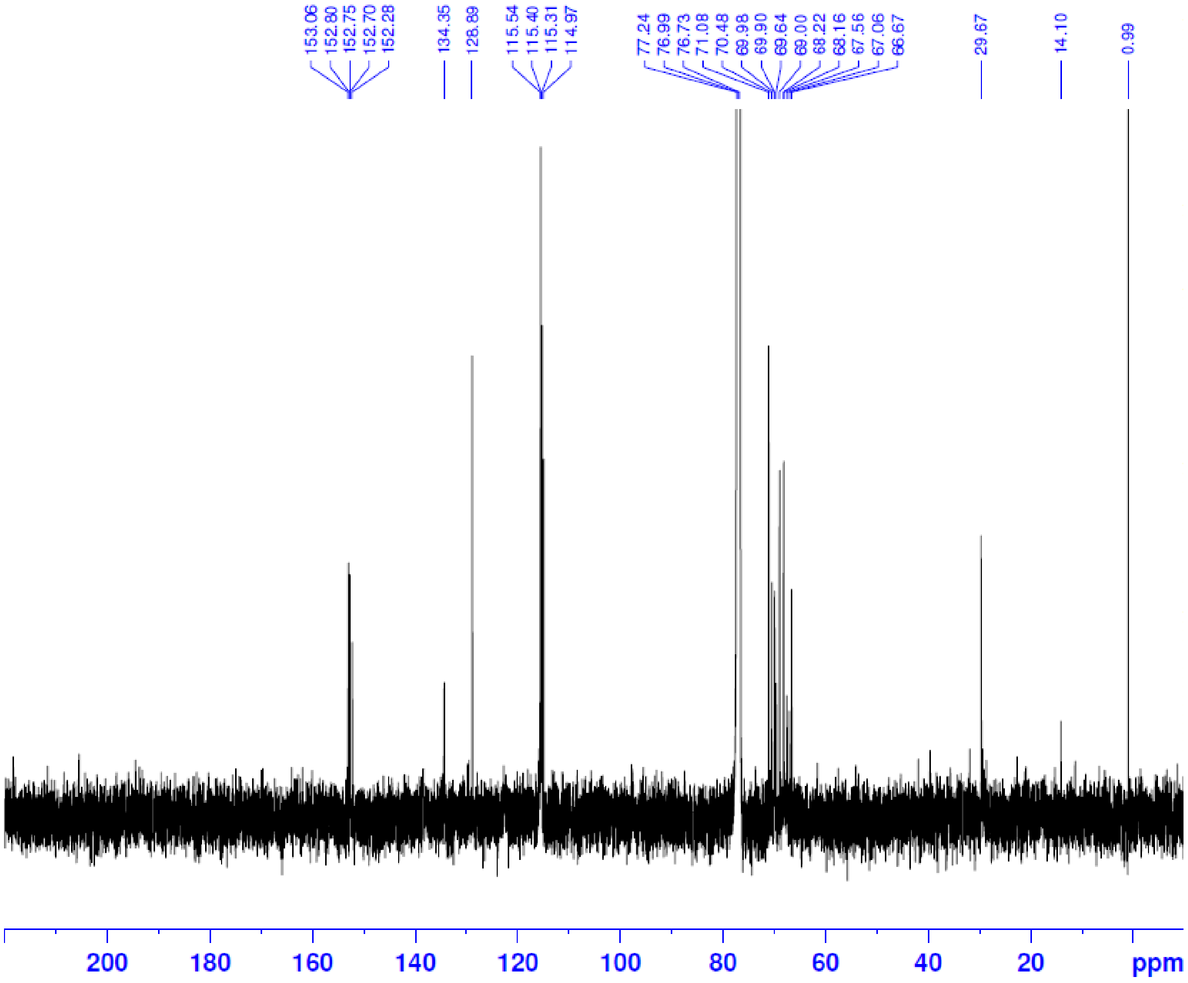

Figure S2: ^{13}C NMR spectrum of catenane **7·BF_4** (125.8 MHz, $CDCl_3$, 298 K).

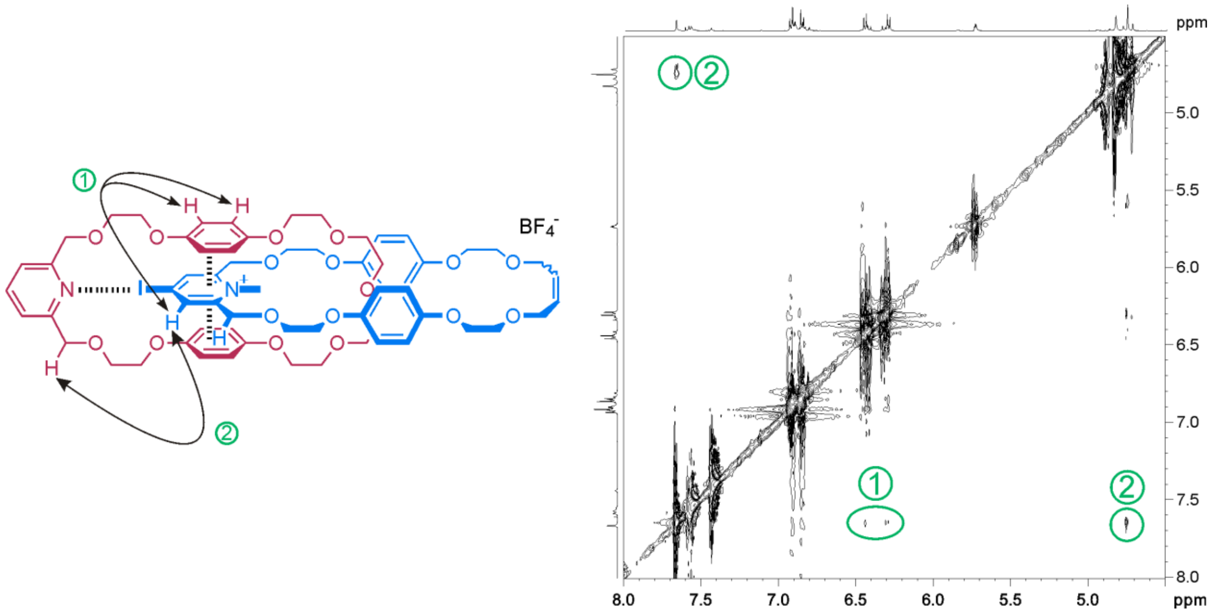

Figure S3: ^{1}H–^{1}H ROESY NMR spectrum of catenane **7·BF$_4$**. Assignable inter-component through-space interactions are highlighted**.**

Part III: Titration Protocols.

^{1}H NMR spectra were recorded on a Varian Unity Plus 500 spectrometer. Typically, a solution of guest was added to a solution of the host at 293 K. The chemical shift of the hydroquinone protons signal of the host was monitored for seventeen titration points (for 0, 0.2, 0.4, 0.6, 0.8, 1.0, 1.2, 1.4, 1.6, 1.8, 2.0, 2.5, 3.0, 4.0, 5.0, 7.0 and 10.0 equivalents of added guest). The resulting data were analysed using the WinEQNMR2 computer program in experiments where association of guest and host was fast on the NMR timescale. Pseudorotaxane titration experiments were carried out using the tetrafluoroborate salt of the threading component guest species (**2·BF$_4$**, **3·BF$_4$**, **4·BF$_4$**, **5·BF$_4$**, **6·BF$_4$)**, titrated into the host species pyridine macrocycle **1**. A 0.139 mol dm^{-3} solution of thread was added to 0.5 mL of a 0.00191 mol dm^{-3} solution of macrocycle. The volumes of thread solution added were 10 x 4 μL, 2 x 10 μL, 2 x 20 μL, 1 x 40 μL, and 1 x 60 μL.

The values of the observed chemical shift and the guest concentration were entered into winEQNMR2 for every titration point, and estimates for the association constant, limiting

chemical shifts and binding stoichiometry made. The parameters were refined using non-linear least squares analysis to obtain the best fit between observed and calculated chemical shifts; the program plots the observed shift versus the guest concentration, revealing the accuracy of the experimental data and the suitability of the model used. The input parameters were varied until the best-fit values of the stability constants, together with their errors, converged.

Part IV: Molecular Modelling.

Computational Details.

DFT calculations and halogen bond parameterization for Molecular Dynamics simulations. The ability of [2]catenane **7** to form C-I···N halogen bonding interactions, was investigated by classical Molecular Dynamics (MD) simulations, using a similar procedure reported by us in references[8] and [9]. The specific force field parameters used to describe these interactions were developed as follows. DFT calculations were performed with Gaussian 09[10] using the hybrid functional B3LYP[11] on systems **A1**, and **B1** (see Figure S4), models for the I···N interaction in $\mathbf{7}^{+}$. System **B1** contains two *meta*-methoxy substituents as mimetic groups of the ether linkages of the two interlocked macrocycles on the [2]catenane assembly.

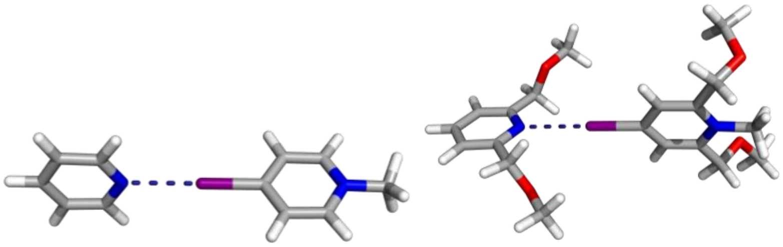

A1 **B1**

Figure S4: Optimised B3LYP gas phase structures of **A1** and **B1** models used in the parameterization of C-I···N charged assisted interactions.

For C, O, H, and N the standard 6-311+G** basis set was used while I was described by the aug-cc-pVDZ-PP basis set and pseudo potential taken from the EMSL Basis Set Exchange website.[12] The geometries were optimized in the gas phase and, for the larger model **B1**, solvent effects ($CHCl_3$) were also considered by means of IEFPCM calculation with radii and non-electrostatic terms for Truhlar and co-workers' SMD solvation model.[13] The relevant structural parameters are collect in Table S1.

Table S1: Optimised B3LYP gas phase relevant distances (Å) for models **A1**, and **B1**. In parenthesis are shown the optimised distances in $CHCl_3$ using a Polarizable Continuum Model.

	**A1**	**B1**	
**C-I**	2.138	2.122	(2.121)
**I···N**	2.720	2.896	(3.061)

It is evident that the halogen bond distance increases slightly with the presence of larger substituents whereas the use of an implicit solvent model indicates that the C-I···N halogen bonds are ~ 3.0 Å long.

Taking this in consideration, we proceeded with the parameterization of the possible C-I···N halogen bond interactions present in **7·BF$_4$**. Parameterizations were performed with the addition of a specific term to the General Amber Force Field (GAFF)[14] parameters used to describe the macrocycle components of the [2]catenane interlocked assembly as suggested in references [15] and [16]. The positive region on the electrostatic potential of the iodopyridinium moiety centred on the iodine atom is represented by a pseudo-atom (DU) with a van de Waals parameter set to zero, a C-I-DU bond angle set to 180º, and a bending force constant of 150 kcal mol^{-1} $Å^{-2}$. The optimal I-DU equilibrium distance was evaluated using several I-DU distances and computing the electrostatic potential and the Restricted Electrostatic Potential (RESP) charges for each new DU position. This was followed by a Molecular Mechanics (MM) minimization of the corresponding assembled catenane structures in Amber 12[17].

Subsequently, the MM optimised distances (I···N) were compared with the DFT data (Table 2). The electrostatic potential for the two individual macrocycles was calculated at the HF/6-31G* level, with the aug-cc-pVDZ-PP basis set for iodine using previously optimized B3LYP geometries at the theory level reported above for the model systems, and the atomic point charges for the individual macrocycles were determined according to the RESP methodology[[18]] using a single conformation and Gaussian IOp (6/42=6) in agreement with the original GAFF reference.[[7]] The MM optimized I···N and C-I distances are reported in Table S2 along with the relevant RESP charges.

Table S2: Gas phase MM optimised distances (Å) as a function of the I-DU distance together with the DU and iodine RESP charges.

I-DU distance	C-I	I···N	DU charge	I charge
**2.00**	2.091	3.118	0.108788	-0.111542
**2.10**	2.092	3.062	0.097845	-0.090483
**2.20**	2.093	2.963	0.088007	-0.070660
**2.35**	2.164	2.600	0.074972	-0.042888
**without DU**	2.092	---	---	0.186479

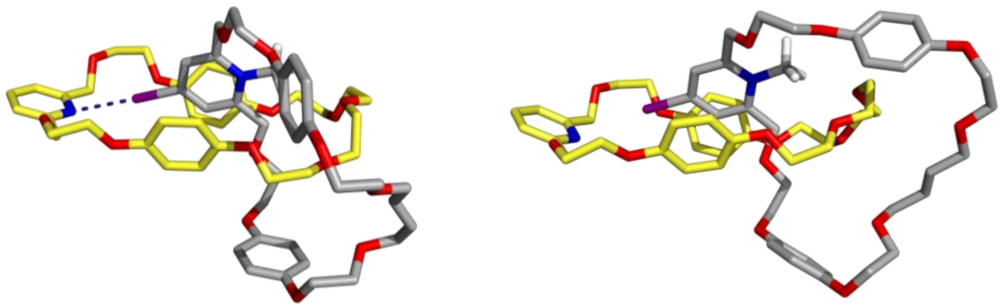

Figure S5: MM minimized interlocked structures of **7**$^{+}$ (top) with DU (I-DU distance 2.10 Å, left) and without DU (right) for the C-I···N halogen bond.

If a DU atom is not used, the halogen bond interactions are lost whereas the addition of the DU atom, independently of the distance, enables the description of the halogen bonds (see Figure S5). However, values of I-DU larger than 2.10 Å are not suitable since atom clashes occur during the subsequent MD simulations performed either in gas phase or in explicit CH_3Cl solution. The I-DU = 2.10 Å leading to an I···N distance of 3.062 Å, which compares well with the 3.061 Å value obtained for the DFT model **B1** in $CHCl_3$ continuum solvent model, is the optimum value to describe I···N interactions and was selected for the further MD simulations reported in this work.

Moreover, preliminary gas phase MM calculations on the iodopyridinium macrocycle showed that default potential energy barrier (V_n) of 0.3 kcal for the X-*ca*-*na*-X torsion angle (where *ca* and *na* are the atom types assigned to the pyridinium ring carbon atoms and nitrogen atom, respectively, and X means any other atom type) led to a slight bending of the *N*-methyl group relatively to pyridinium ring plane. This structural imprecision was corrected using the torsion force field parameters available in the GAFF for the related X-*cd*-*na*-X torsion angle (4 / 6.8 / 180.0 / 2), where *cd* is a sp^2 carbon in non-pure aromatic systems.

Generation of starting structures for the MD simulations. Since there is no X-ray structure for catenane **7·BF$_4$** and given its conformational freedom, the starting structures for the MD simulations were generated by a protocol based on a gas-phase quenched dynamics simulation.[19] The previous MM optimised structure with I-DU = 2.10 Å was subjected to a 5 ns MD run at 500 K saving a trajectory with 10000 structures that should sample most of the conformational space. All these structures were minimized by MM and clustered by RMSD similarity. Two structures corresponding to representative conformations of the most populated clusters were selected. These structures shown in Figure S6 as **S1A** and **S1B** present an C-I···N halogen bond and π–π donor-acceptor interactions between the

iodopyridinium and the hydroquinone rings of the macrocycle **1**. These two co-conformations basically differ in the conformation adopted by the iodopyridinium macrocycle leading to slightly different binding scenarios.

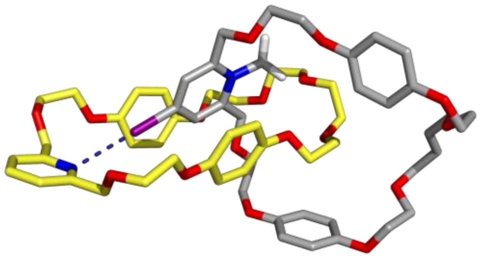

S1A

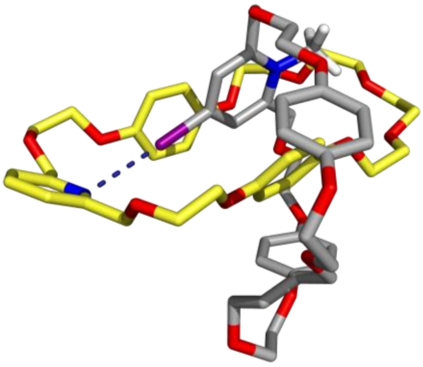

S1B

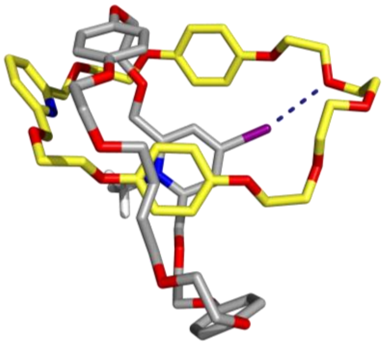

S2A

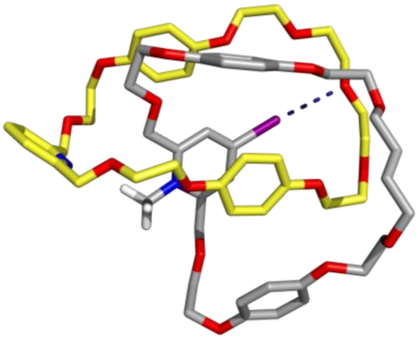

S2B

Figure S6: Starting co-conformations used in the MD simulations, **S1A** and **S1B** exhibit an C-I···N halogen bond and **S2A** and **S2B** present an C-I···O halogen bond.

In order to increase the sampling, the iodopyridinium macrocycle in a structure similar to **S1A** was rotated affording an interlocked assembly with the C-I bond pointing away from the N of the pyridine ring. This struture was subjected to the same protocol mentioned earlier yielding two starting co-conformations **S2A** and **S2B** presented in Figure S6, where the C-I bond is pointing towards an oxygen atom from the polyether loop of the macrocycle **1**.

Molecular Dynamics Simulations. The MM minimized conformations shown in Figures S6 were immersed in cubic box of $CHCl_3$ molecules[20] (~ 43 Å side, after equilibration). One BF_4^- anion, with charges and parameters from reference[21], was used as counter-ion. All systems were simulated using the following multi-stage protocol. The solvent was initially relaxed keeping the solute fixed with a strong positional restraint (500 kcal mol^{-1} $Å^{-2}$). Then, the restraint was removed and a MM energy minimisation of entire system was performed followed by a heating stage (0 to 300 K) during 50 ps using the Langevin thermostat with a collision frequency of 1 ps^{-1} in an NVT ensemble and a weak positional restraint on the solute (10 kcal mol^{-1} $Å^{-2}$). The system was then equilibrated for 5 ns in a NPT ensemble at 1 atm with isotropic pressure scaling using relaxation time of 2 ps and data collection was performed for 50 ns. All simulations were carried out with the pmemd.cuda AMBER executable, able to accelerate explicit solvent Particle Mesh Ewald (PME) calculations[22] trough the use of GPUs.[23] All bonds involving hydrogen atoms were constrained using SHAKE[24] allowing the usage of 2 fs time step. A 10 Å cutoff was used for the non-bonded van der Waals interactions. Trajectory analysis was performed with the cpptraj utility of AmberTools12 while the molecular representations were drawn with PyMOL.[25] Plots were performed with Gnuplot.[26] Representative co-conformations from the MD trajectories in solution were obtained by clustering the structures by RMSD similarity, using the average-linkage clustering algorithm.[27] A frame from the most populated cluster was chosen as representative of the simulation.

MD simulations extended analysis.

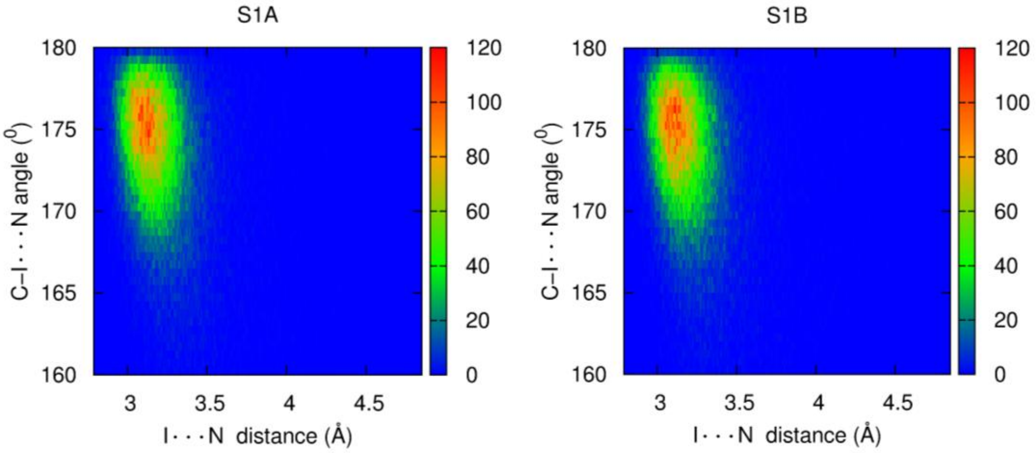

Figure S7: Histograms for the distribution of the I···N distances (Å) and the C-I···N angles (º) for simulations with **S1A** and **S1B**. Data were collected during 50 ns.

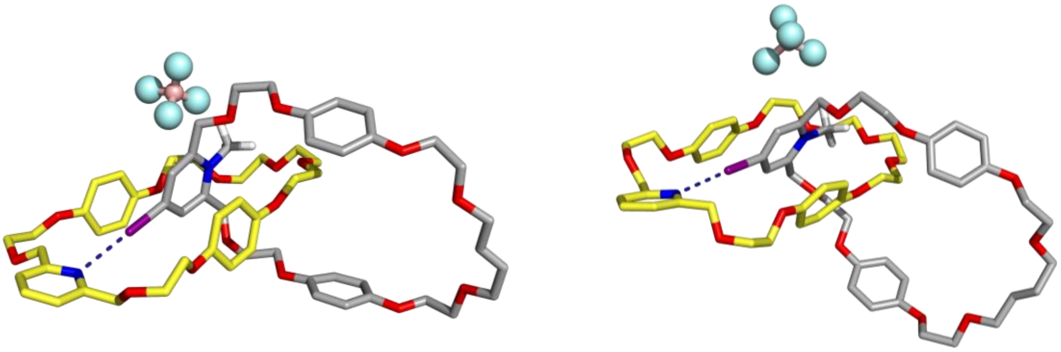

Figure S8: Representative co-conformations of **7·BF$_4$** for simulations with **S1A** (left) and **S1B** (right).

Extended discussion on simulations with S2A and S2B. In the **S2** scenarios (**A** and **B**), an C-I···O halogen bond is possible between the iodopyridinium and the three available oxygen atoms of the polyether loop numbered O1, O2, and O3 (see Figure S9). In these simulations we also monitored the distribution of the I···O distances and C-I···O angles and the results are plotted in Figure S9. It is clear that the results are very similar and independent of the starting co-conformation (**A** or **B**). Concerning the interaction with O1 or O3, the distances are too long and the angles are too low to be considered halogen bonds. For the C-I interaction with O2, the values are distributed around values more compatible with the presence of an halogen bond with average I···O distances and C-I···O angles of 3.60 Å and 164º for **A** and 3.51 Å and

164º for **B** co-conformations. However, contrary to what was found for simulations of C-I···N halogen bonds in **S1A** and **S1B**, the distributions of I···O distances for the simulations with **S2A** and **S2B** structures are very diffuse with respect to the C-I···O angle as evident when the corresponding histograms for this two type of halogen bonds are compared (Figure S7 *versus* Figure S9 middle. This is an indication that the possible C-I···O halogen bond is not sufficiently stable for halogen bond templation as it is the charged assisted I···N halogen bond.

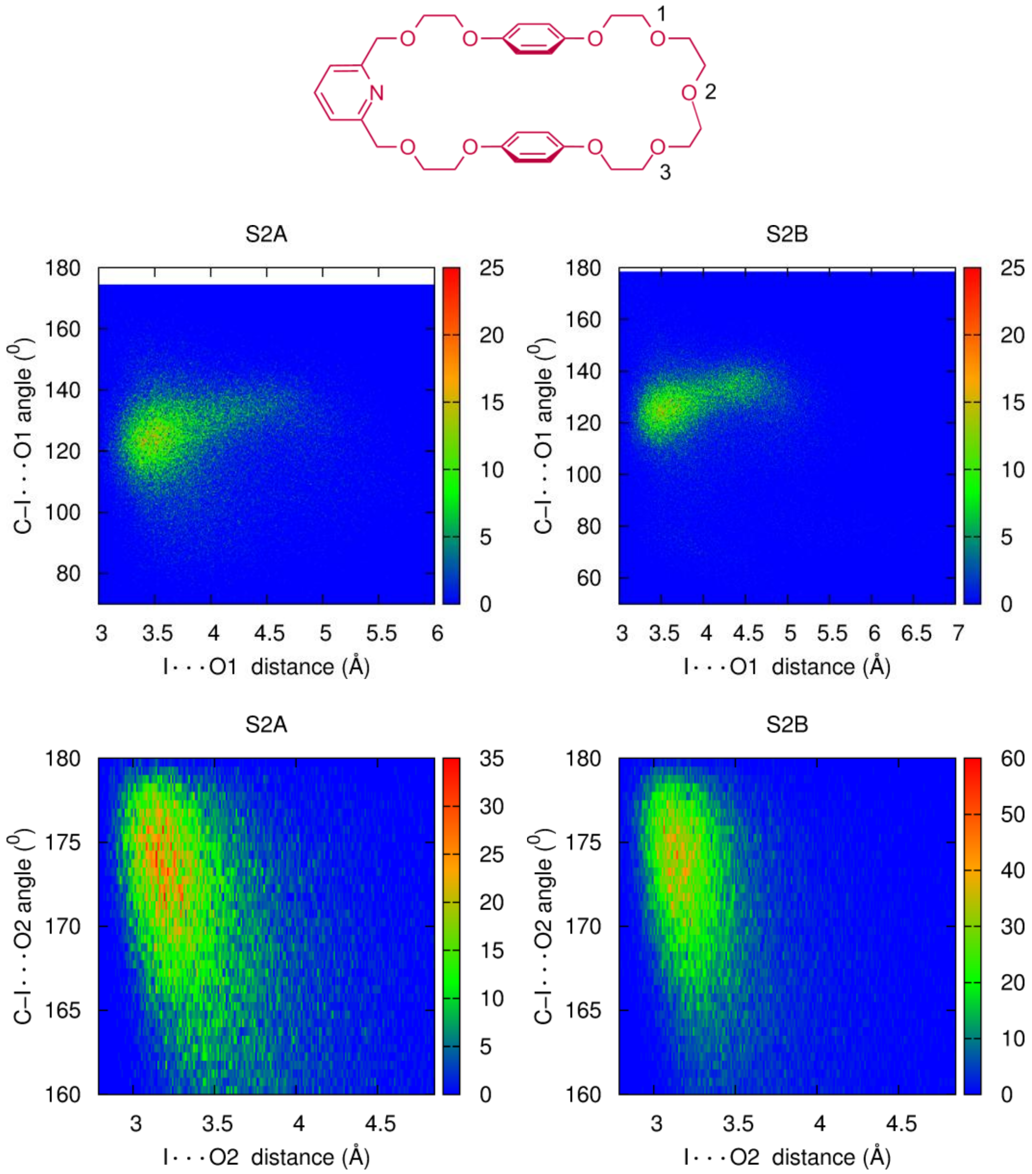

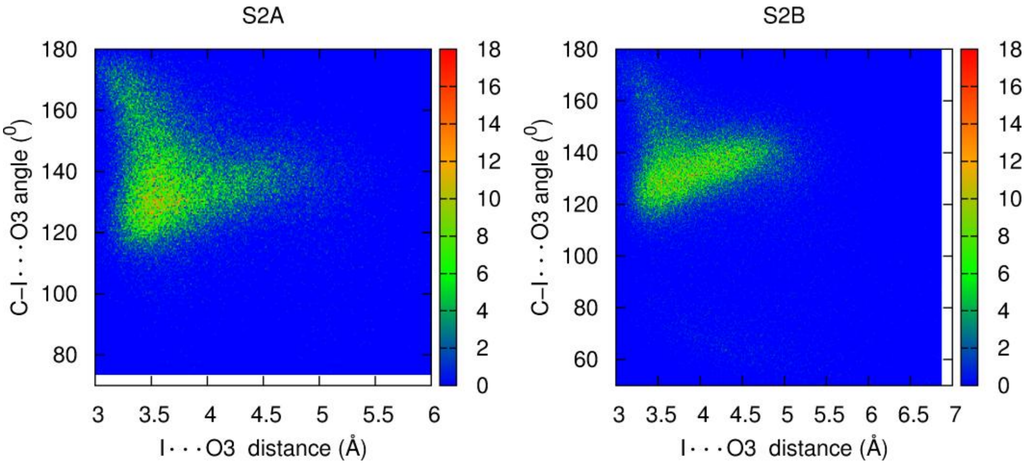

Figure S9: Histograms for the distribution of the I···O distances (Å) and the C-I···O angles (º) for simulations with **S2A** and **S2B**. Data were collected along the 50 ns. The numbering scheme used for oxygen atoms is shown at the top.

Part V: References.

[1] K. M. Bonger, R. J. B. H. N. van den Berg, L. H. Heitman, A. P. Ijzerman, J. Oosterom, C. M. Timmers, H. S. Overkleeft, G. A. van der Marel, *Biorg. Med. Chem.* **2007**, *15*, 4841-4856.
[2] M. D. Lankshear, N. H. Evans, S. R. Bayly, P. D. Beer, *Chem. Eur. J.* **2007**, *13*, 3861-3870.
[3] S. Bhattacharya, K. Snehalatha, V. P. Kumar, *J. Org. Chem.* **2003**, *68*, 2741-2747.
[4] H. Takalo, P. Pasanen, J. Kankare, *Acta Chem. Scand., Ser. B* **1988**, *42*, 373-377.
[5] P. Roquette, C. König, O. Hübner, A. Wagner, E. Kaifer, M. Enders, H.-J. Himmel, *Eur. J. Inorg. Chem.* **2010**, *2010*, 4770-4782
[6] Crombie, L.; Ryan, A. P.; Whiting, D. A.; Yeboah, S. O. *J. Chem. Soc., Perkin Trans. 1* **1987**, 2783-2786.
[7] Choudhury, M. K. *J. Chem. Res., Synop* . **1995**, 157
[8] A. Caballero, F. Zapata, N. G. White, P. J. Costa, V. Félix, P. D. Beer, *Angew. Chem. Int. Ed.* **2012**, *51*, 1876–1880.
[9] F. Zapata, A. Caballero, N. G. White, T. D. W. Claridge, P. J. Costa, V. Félix, P. D. Beer, *J. Am. Chem. Soc.* **2012**, *134*, 11533−11541.
[10] Gaussian 09, M. J. Frisch, G. W. Trucks, H. B. Schlegel, G. E. Scuseria, M. A. Robb, J. R. Cheeseman, J. J. A. Montgomery, T. Vreven, K. N. Kudin, J. C. Burant, J. M. Millam, S. S. Iyengar, J. Tomasi, V. Barone, B. Mennucci, M. Cossi, G. Scalmani, N. Rega, G. A. Petersson, H. Nakatsuji, M. Hada, M. Ehara, K. Toyota, R. Fukuda, J. Hasegawa, M. Ishida, T. Nakajima, Y. Honda, O. Kitao, H. Nakai, M. Klene, X. Li, J. E. Knox, H. P. Hratchian, J. B. Cross, V. Bakken, C. Adamo, J. Jaramillo, R. Gomperts, R. E. Stratmann, O. Yazyev, A. J. Austin, R. Cammi, C. Pomelli, J. W. Ochterski, P. Y. Ayala, K. Morokuma, G. A. Voth, P. Salvador, J. J. Dannenberg, V. G. Zakrzewski, S. Dapprich, A. D. Daniels, M. C. Strain, O. Farkas, D. K. Malick, A. D. Rabuck, K. Raghavachari, J. B. Foresman, J. V. Ortiz, Q. Cui, A. G. Baboul, S. Clifford, J. Cioslowski, B. B. Stefanov, G. Liu, A. Liashenko, P. Piskorz, I. Komaromi, R. L. Martin, D. J. Fox, T. Keith, M. A. Al-Laham, C. Y. Peng, A. Nanayakkara, M. Challacombe, P. M. W. Gill, B. Johnson, W. Chen, M. W. Wong, C. Gonzalez, J. A. Pople, Gaussian, Inc., Wallingford CT, **2009**.
[11] a) A. D. Becke, *J. Chem. Phys.* **1993**, *98*, 5648-5652; b) C. Lee, W. Yang, R. G. Parr, *Phys. Rev. B* **1988**, *37*, 785-789.
[12] K. L. Schuchardt, B. T. Didier, T. Elsethagen, L. Sun, V. Gurumoorthi, J. Chase, J. Li, T. L. Windus, *J. Chem. Inf. Model.* **2007**, *47*, 1045-1052.
[13] A. V. Marenich, C. J. Cramer, D. G. Truhlar, *J. Phys. Chem. B* **2009**, *113*, 6378-6396.
[14] J. Wang, M. R. Wolf, J. W. Caldwell, P. A. Kollman, D. A. Case, *J. Comput. Chem.* **2004**, *25*, 1157.
[15] M. A. A. Ibrahim, *J. Comput. Chem.* **2011**, *32*, 2564–2574.
[16] S. Rendine, S. Pieraccini, A. Forni, M. Sironi, *Phys. Chem. Chem. Phys.* **2011**, *13*, 19508–19516.
[17] D. A. Case, T. A. Darden, T. E. Cheatham, III, C. L. Simmerling, J. Wang, R. E. Duke, R. Luo, R. C. Walker, W. Zhang, K. M. Merz, B. Roberts, S. Hayik, A. Roitberg, G. Seabra, J. Swails, A. W. Götz, I. Kolossváry, K. F. Wong, F. Paesani, J. Vanicek, R. M. Wolf, J. Liu, X. Wu, S. R. Brozell, T. Steinbrecher, H. Gohlke, Q. Cai, X. Ye, J. Wang, M.-J. Hsieh, G. Cui, D. R. Roe, D. H. Mathews, M. G. Seetin, R. Salomon-

Ferrer, C. Sagui, V. Babin, T. Luchko, S. Gusarov, A. Kovalenko, P. A. Kollman, AMBER 12, University of California, San Francisco, 2012.
[18] C. I. Bayly, P. Cieplak, W. Cornell, P. A. Kollman, *J. Phys. Chem.* **1993**, *97*, 10269-10280.
[19] A. Rappe, C. Casewit, "*Molecular Mechanics Across Chemistry*", University Science Books, Sausalito, CA, **1997**.
[20] P. Cieplak, J. Caldwell, P. Kollman, *J. Comput. Chem.*, **2001**, *22*, 1048–1057.
[21] X. Wu, Z. Liu, S. Huang, W. Wang, *Phys. Chem. Chem. Phys*. **2005**, *7*, 2771-2779.
[22] a) T. Darden, D. York, L. Pedersen, *J. Chem. Phys.* **1993**, *98*, 10089–10092; b) U. Essmann, L. Perera, M. L. Berkowitz, T. Darden, H. Lee, L. G. Pedersen, *J. Chem. Phys.* **1995**, *103*, 8577–8593.
[23] a) see http://ambermd.org/gpus/ (accessed in 2012/10/10). b) A. W. Goetz, R. Salomon-Ferrer, D. Poole, S. L. Grand, R. C. Walker, "Routine microsecond molecular dynamics simulations with AMBER - Part II: Particle Mesh Ewald" (in preparation), **2011**.
[24] J.-P. Ryckaert, G. Ciccotti, H. J. C. Berendsen, *J. Comput. Phys.* **1977**, *23*, 327–341.
[25] PyMOL Molecular Graphics System, Version 1.2r2, DeLano Scientific LLC, **2009**.
[26] T. Williams, C. Kelley, Gnuplot 4.4: An Interactive Plotting Program, **2010**. URL: http://www.gnuplot.info
[27] J. Shao, S. W. Tanner, N. Thompson, T. E. Cheatham III, *J. Chem. Theory Comput.* **2007**, *3*, 2312–2334.
